# Synthesizing and Salvaging NAD^+^: Lessons Learned from *Chlamydomonas reinhardtii*

**DOI:** 10.1371/journal.pgen.1001105

**Published:** 2010-09-09

**Authors:** Huawen Lin, Alan L. Kwan, Susan K. Dutcher

**Affiliations:** 1 Department of Genetics, Washington University School of Medicine, St. Louis, Missouri, United States of America; 2 Department of Computer Science and Engineering, Washington University in St. Louis, St. Louis, Missouri, United States of America; Stanford University, United States of America

## Abstract

The essential coenzyme nicotinamide adenine dinucleotide (NAD^+^) plays important roles in metabolic reactions and cell regulation in all organisms. Bacteria, fungi, plants, and animals use different pathways to synthesize NAD^+^. Our molecular and genetic data demonstrate that in the unicellular green alga *Chlamydomonas* NAD^+^ is synthesized from aspartate (*de novo* synthesis), as in plants, or nicotinamide, as in mammals (salvage synthesis). The *de novo* pathway requires five different enzymes: L-aspartate oxidase (ASO), quinolinate synthetase (QS), quinolate phosphoribosyltransferase (QPT), nicotinate/nicotinamide mononucleotide adenylyltransferase (NMNAT), and NAD^+^ synthetase (NS). Sequence similarity searches, gene isolation and sequencing of mutant loci indicate that mutations in each enzyme result in a nicotinamide-requiring mutant phenotype in the previously isolated *nic* mutants. We rescued the mutant phenotype by the introduction of BAC DNA (*nic2-1* and *nic13-1*) or plasmids with cloned genes (*nic1-1* and *nic15-1*) into the mutants. NMNAT, which is also in the *de novo* pathway, and nicotinamide phosphoribosyltransferase (NAMPT) constitute the nicotinamide-dependent salvage pathway. A mutation in NAMPT (*npt1-1*) has no obvious growth defect and is not nicotinamide-dependent. However, double mutant strains with the *npt1-1* mutation and any of the *nic* mutations are inviable. When the *de novo* pathway is inactive, the salvage pathway is essential to *Chlamydomonas* for the synthesis of NAD^+^. A homolog of the human *SIRT6*-like gene, *SRT2*, is upregulated in the NS mutant, which shows a longer vegetative life span than wild-type cells. Our results suggest that *Chlamydomonas* is an excellent model system to study NAD^+^ metabolism and cell longevity.

## Author Summary

Nicotinamide adenine dinucleotide (NAD^+^) is an essential coenzyme. NAD^+^ is necessary for electron transfer in many metabolic reactions. NAD^+^ functions as a substrate for several enzymes, one of which is sirtuin, an enzyme involved in gene regulation and aging. NAD^+^ can be synthesized either from amino acids (*de novo*) or metabolites (salvage). Given the importance of NAD^+^, enzymes involved in NAD^+^ synthesis are targets for drug discovery. In the unicellular green alga *Chlamydomonas* we investigated both the *de novo* and salvage NAD^+^ biosynthetic pathways. Mutations in the plant-like *de novo* synthesis pathway lead to a nicotinamide-requiring phenotype. We identified an insertional mutation in the first enzyme in the mammal-like salvage pathway; it has no growth defect in cells with an active *de novo* synthesis pathway but causes lethality when the *de novo* synthesis pathway is inactive. Coupled with NAD^+^ biosynthesis, sirtuin is involved in NAD^+^ consumption. Our study links upregulation of a sirtuin gene with extended life span in the *nic13-1* mutant strain, which has a defective *de novo* synthesis pathway and suggests that *Chlamydomonas* is an excellent genetic model to study NAD^+^ metabolism and cell longevity.

## Introduction

The coenzyme nicotinamide adenine dinucleotide (NAD^+^) is an essential enzyme. Electron transfer between NAD^+^ and its reduced form NADH are essential to cells as they are involved in glycolysis and the citric acid cycle as well as regeneration of ATP from ADP [1]. NAD^+^ is consumed in several non-redox processes in cells (see [2] for review). NAD^+^ is a substrate of ADP-ribosyl transferase, which transfers ADP-ribose from NAD^+^ to ADP-ribose receptors, which are involved in DNA damage responses, transcriptional regulation, chromosome separation and apoptosis. NAD^+^ is also the target of ADP-ribosyl cyclases, which produce cyclic ADP-ribose that acts in second messenger signaling pathways. NAD^+^ is a substrate of sirtuins (SIRT/Sir2, Silent Information Regulator Two), a group of NAD^+^-dependent deacetylases that remove acetyl groups from lysine residues on histones, microtubules, and other proteins. Thus, NAD^+^, via sirtuins, modulates many events.

**Figure 1:**
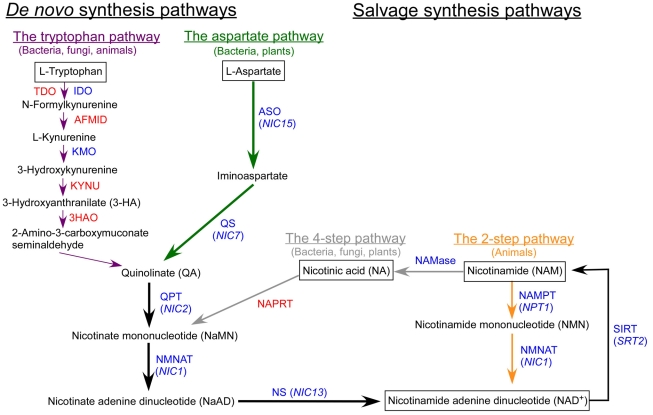
The biosynthetic pathways of nicotinamide adenine dinucleotide (NAD^+^). Enzymes that are present in *Chlamydomonas* are indicated in blue and enzymes that are absent are indicated in red. Green arrows indicate steps specific to the aspartate pathway; dark purple arrows indicate steps specific to the tryptophan pathway; gray arrows indicate steps specific to the 4-step pathway; orange arrows indicate steps specific to the 2-step pathway; black arrows indicate steps that are commonly shared by multiple pathways. Abbreviations: ASO, L-Aspartate oxidase; QS, Quinolinate synthetase; QPT, Quinolinate phosphoribosyltransferase; NMNAT, Nicotinate/nicotinamide mononucleotide adenylyltransferase; NS, NAD^+^ synthetase; TDO, Tryptophan 2,3-dioxygenase; IDO: Indoleamine 2,3-dioxygenase; AFMID, Arylformamidase; KMO, Kynurenine 3-monooxygenase; KYNU, Kynureninase; 3HAO, 3-Hydroxy- anthranilate 3,4-dioxygenase; NAMase, nicotinamidase; NAPRT, nicotinate phosphoribosyltransferase; NAMPT, nicotinamide phosphoribosyltransferase; SIRT, silent information regulator two. *Chlamydomonas* genes identified in this study are indicated in parentheses.

NAD^+^ synthesis pathways are categorized into either *de novo* pathways, which start with the amino acid aspartate or tryptophan, or salvage pathways, which start with nicotinamide (NAM) or nicotinic acid (NA) (Figure 1). Plants and some bacteria initiate *de novo* synthesis from aspartate and use two enzymes, L-aspartate oxidase (ASO) and quinolinate synthetase (QS), to synthesize quinolinate (QA). Fungi, animals and some bacteria synthesize QA from tryptophan via six enzymes, tryptophan 2,3-dioxygenase (TDO)/indoleamine 2,3-dioxygenase (IDO), arylformamidase (AFMID), kynurenine 3-monooxygenase (KMO), kynureninase (KYNU), and 3-hydroxy-anthranilate 3,4-dioxygenase (3HAO). The three enzymes shared by both *de novo* pathways, quinolinate phosphoribosyltransferase (QPT); nicotinate/nicotinamide mononucleotide adenylyltransferase (NMNAT); and NAD synthetase (NS), are required for the conversion of QA to NAD^+^.

In the salvage pathways, the starting substrate is usually NA or NAM (Figure 1). Fungi, plants, and most bacteria, use NAM in a 4-step process involving nicotinamidase (NAMase), nicotinate phosphoribosyltransferase (NAPRT), NMNAT, and NS to synthesize NAD^+^. In *C. elegans*, this is the only known pathway to synthesize NAD^+^ [3]. On the other hand, in mammals and some bacteria, NAD^+^ is synthesized via a 2-step enzymatic process and the enzymes involved are nicotinamide phosphoribosyltransferase (NAMPT) and NMNAT.

The consumption of NAD^+^ by sirtuin mediated-protein deacetylation results in the production of nicotinamide. Recent studies have linked SIRT proteins to transcriptional gene silencing [4], DNA break repair [5], cell cycle regulation [6], aging [7], metabolism [8] and apoptosis [9]. In human, seven members of the SIRT protein family, SIRT1-7, are separated into 5 classes, I-IV, and U [10]. Human SIRT6, SIRT7, and some plant SIRT proteins belong to Class IV [10], [11]. The nuclear-localized SIRT6 is a NAD^+^-dependent histone deacetylase involved in telomeric chromatin modulation [12]. Deficiency of *SIRT6* in mice is correlated with defective DNA repair, genomic instability, age-related degeneration [7], as well as increased glucose uptake, which is caused by transcriptional upregulation of several glycolytic genes that are normally repressed by SIRT6 [8]. SIRT7 localizes to the nucleolus and is involved in gene regulation of rDNA [13], [14].

*Chlamydomonas reinhardtii*, a unicellular green alga, is evolutionarily related to the seed plants and contains a chloroplast [15], [16]. Additionally, it contains animal specific organelles known as cilia/flagella and centrosomes. As discussed above, NAD^+^ synthesis pathways are diverse, but enzymes involved at each specific step are conserved in many organisms. Sequence similarity searches indicate that enzymes involved in the aspartate pathway from *Arabidopsis*, rice, and *E. coli* are conserved with protein identities ranging from 22% to 70% [17]. With the completion of the *Chlamydomonas* genome project [15], it became possible to identify *Chlamydomonas* homologs involved in the NAD^+^ synthesis pathways via sequence similarity searches.

A group of NAM-requiring mutants (*nic*) was isolated by Eversole that fail to grow well on medium lacking NAM [18]. The mutations also confer sensitivity to 3-acetylpyridine (3-AP) [19]. Eight NAM-requiring strains were originally isolated and six of these mutant strains are still extant. The *NIC7* locus was identified in a walk through the mating-type locus and shown to encode a homolog of QS [20], [21]. We tested whether the remaining *NIC* loci encode the enzymes of the *de novo* aspartate NAD^+^ synthesis pathway. The *nic* mutant loci define six different loci and map to six different linkage groups (LG) [19]: *NIC1* maps to LG XV; *NIC2* maps to LG II; *NIC7* maps to LG VI; *NIC11* maps to LG IV; *NIC13* maps to LG X; and *NIC15* maps to LG XII/XIII [[20], [ 22]–[25]; see Materials and Methods for linkage group to chromosome translation]. In our study, phenotypic characterization and genetic crosses of *nic11* strains obtained from the *Chlamydomonas* Center indicate that the Nic^−^ phenotype of these *nic11* strains (sensitivity to 3-AP or a growth defect on medium lacking NAM) can no longer be scored. Therefore, only five mutant strains are used in our study and we find that they encode the five enzymes in the *de novo* biosynthesis of NAD^+^ from aspartate.

## Results

### *Chlamydomonas* Nic^−^ mutant phenotype can be rescued by addition of NAM

**Figure 2:**
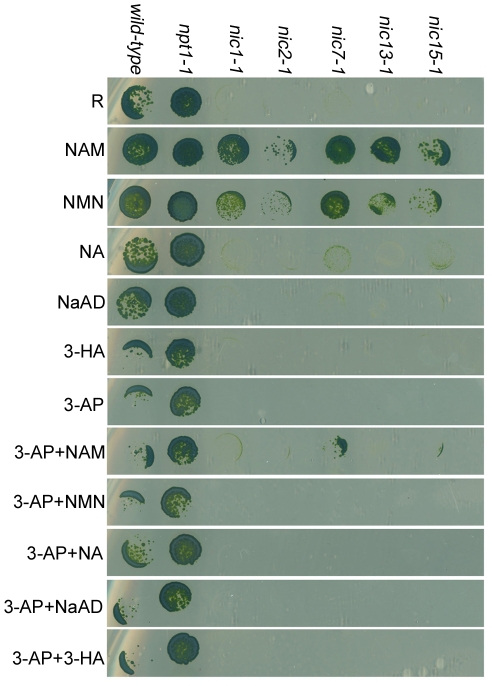
*Chlamydomonas* nicotinamide-requiring *nic* mutants show 3-AP sensitivity. *Chlamydomonas* cells were spotted on solid rich medium (R) or medium supplemented with 10 µM NAM (nicotinamide), 10 µM NMN (nicotinamide mononucleotide), 10 µM NA (nicotinic acid), 10 µM NaAD (nicotinate adenine dinucleotide), or 10 µM 3-HA (3-hydroxyanthranilate). For 3-AP (3-acetylpyridine) sensitivity assay, cells were spotted on R medium containing 16.5 mg/l 3-AP with or without the addition of various chemical substances as indicated.

Wild-type (CC-124) and five different *nic* mutant cells (*nic1-1*, *nic2-1*, *nic7-1*, *nic13-1*, and *nic15-1*) were tested for their ability to utilize intermediate substrates in different NAD^+^ biosynthesis pathways (Figure 2). Wild-type cells show no obvious growth defect on any of the media tested. All the *nic* mutant strains fail to grow on Sager and Granick rich medium without NAM (R) or R medium supplemented with 3-AP, as previously described [19]. These mutants grow well on media supplied with either NAM or NMN, two chemical substances found only in the 2-step salvage biosynthesis pathway of NAD^+^. Addition of NA, an intermediate substrate found in the 4-step salvage pathway showed very weak rescue of the Nic^−^ mutant phenotype of the mutants. Addition of 3-HA, which is synthesized in the tryptophan *de novo* pathway could not rescue the growth defect of any *nic* strains. NaAD, which acts in both *de novo* pathways, also does not rescue the Nic^−^ mutant phenotype. The failure to rescue may indicate a failure of *Chlamydomonas* to transport these metabolites into cells.

3-AP is considered to be a NA analogue, which causes NA deficiency in mice. Injecting animals with NA, NAM, or tryptophan rescues the NA deficiency [26], [27]. Given that 3-AP causes cell lethality in *Chlamydomonas nic* mutant cells, we tested whether addition of NAM or NA could rescue the phenotype. Addition of NAM showed weak rescue of the *nic7-1* and *nic15-1* mutants but not the other mutants (Figure 2). Addition of NA, NMN, NaAD, or 3-HA does not rescue the lethality conferred by 3-AP in any of the mutants.

### *De novo* biosynthesis of NAD^+^ in *Chlamydomonas* resembles the pathway used by seed plants

Katoh *et al.* showed that *Arabidopsis* synthesizes NAD^+^ from aspartate and all five enzymes involved in this pathway have been characterized [28], [29]. We identified the *Chlamydomonas* homologs by sequence similarity and linked the genes to corresponding *nic* mutants via DNA sequencing and complementation with transgenes. The results are summarized in Table 1.

**Table 1:** List of *Chlamydomonas* enzymes involved in *de novo* NAD^+^ biosynthesis from L-aspartate.

EC number	Enzyme	BLAST protein	Chromosome	GenBank Accession number	Identity/similarity	Mutant	Mutation	Gene rescue
1.4.3.16	ASO	NP_568304	125123323–5126728	XP_001696864	51%/65%	*nic15-1*	S_459_F	BAC (n = 14)Plasmid (n = 24)
2.5.1.72	QS	NP_199832	6339304–346,672	HM061643	63%/80%	*nic7-1*	L_351_P	BAC and Plasmid^1^
2.4.2.19	QPT	NP_973393	2909855–9101443	XP_001694961	55%/69%	*nic2-1*	Frame shift at 187, stop codon at 240	BAC (n = 8)
2.7.7.18/2.7.7.1	NMNAT	NP_200392	142771115–2768696	FJ944016	52%/68%	*nic1-1*	Q_345_H, Q_346_stop	BAC (n = 5)Plasmid (n = 2)
6.3.5.1	NS	At1g55090	102338814–2351247	FJ944017	52%/63%	*nic13-1*	S_740_I	Reversion analysis

1 Results from [20].

**Figure 3:**
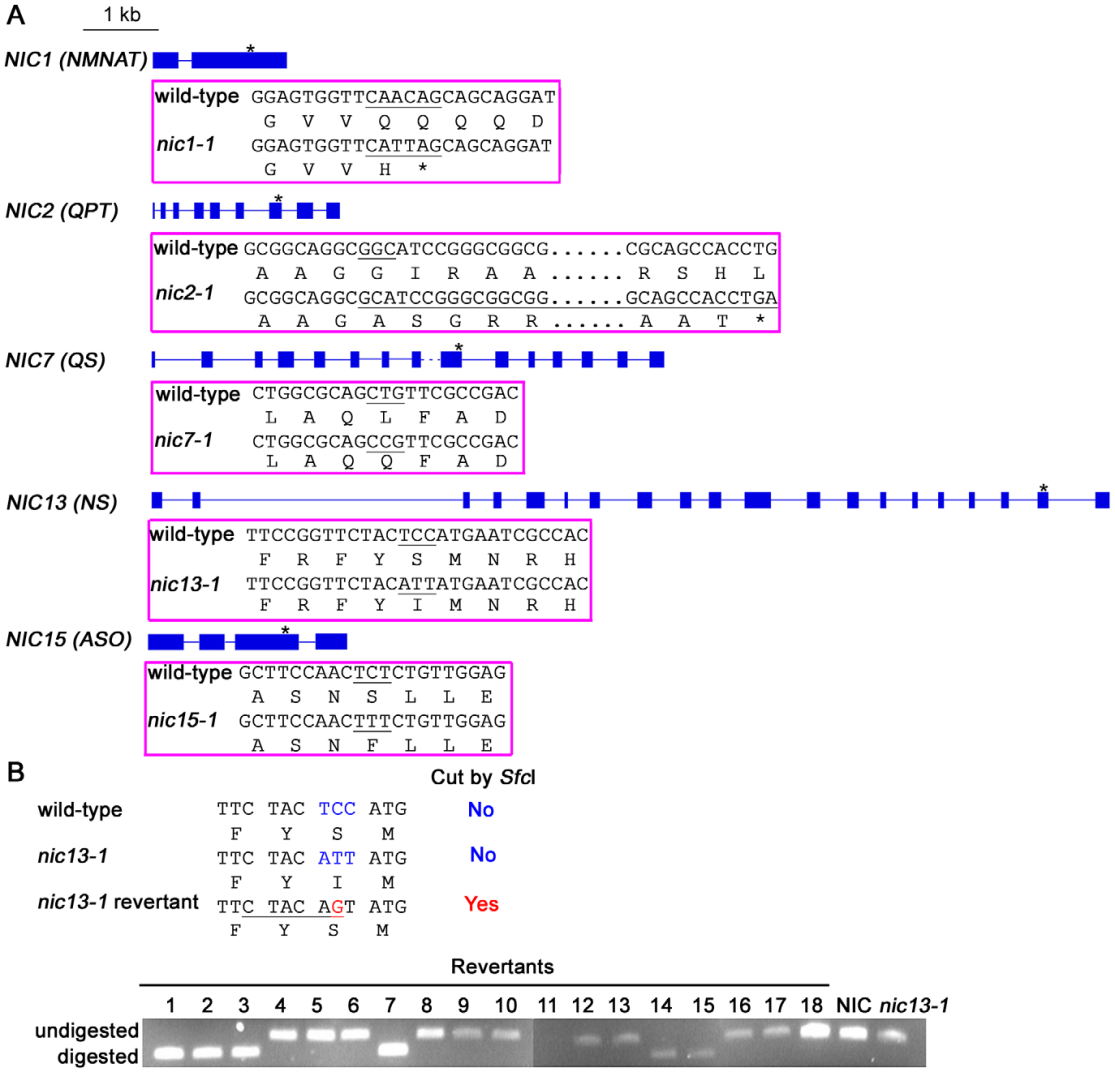
*Chlamydomonas nic* mutants carry various point mutations in *NIC* genes. (A) Schematic diagrams show the gene structures of *NIC1*, *NIC2*, *NIC7*, *NIC13*, *NIC15* and the positions of mutations are indicated by asterisks. Blue solid box, exon; solid line, intron; dashed line, undefined length of intron. Each magenta box below the gene structure indicates changes in nucleotide/amino acid in the mutants when compared to the wild-type strain. Changes in codon that results in amino acid changes are highlighted in black. (B) PCR and enzymatic digestion to identify *nic13-1* I_740_S reversion from 18 *nic13-1* UV revertants. Top, nucleotide and amino acid sequences around the mutation points in various strains. The *Sfc*I restriction enzyme recognition site is underlined in the *nic13-1* revertant sequence. Bottom, *SfcI* digestion products in various strains.

*Chlamydomonas ASO* gene, which contains 4 exons and encodes a 669 aa protein (Figure 3A; [15]), is ∼63 kb away from the *ODA12* gene [30], which maps to LG XII/XIII [31]. The *Chlamydomonas nic15-1* mutant is tightly linked to *MAA1* on LGXII/XIII [22]. The *nic15-1* strain (See Materials and Methods) contains a single nucleotide change C_1376_T that predicts a S_459_F change in the predicted protein (Figure 3A). The S_459_F change falls in a highly conserved region among bacterial and plant ASO proteins and is likely to affect normal function of this protein (Figure S1). We performed gene complementation with either a BAC (32L22) or a plasmid containing the full-length *ASO* gene (pNIC15a). Both transformations produced 3-AP-resistant colonies (n = 14 for the BAC and n = 24 for the plasmid), which indicates that Nic^−^ mutant phenotype was rescued by re-introducing the *ASO* gene.

The *nic7-1* mutant maps to a 7.9 kb region on LG VI. Transformation of this fragment rescues the 3-AP sensitive phenotype of *nic7-1* cells, but the nature of this mutant and the gene structure of *NIC7* were not determined [20]. Ferris *et al.* proposed that *NIC7* encodes QS, given the *NIC7* gene product displays low similarity to bacterial QS [21]. Using RT-PCR and DNA sequencing, we found that the *NIC7* gene contains 15 exons and it shares 63% identity to *Arabidopsis* QS. Sequencing of the *NIC7* coding region reveals a L_351_Q change in the *nic7 -1* mutant (Figure 3A). The amino acid L_351_ within the quinolinate synthetase domain is conserved among almost all plant QS proteins but not in bacterial proteins (Figure S2).

*CNA43*, a cDNA marker mapped to LG II [31] is ∼212kb from the *Chlamydomonas QPT* gene as determined by the JGI *Chlamydomonas* v4.0 genome assembly. The *nic2-1* mutant maps to LG II ([24]; M. Miller and S. K. Dutcher, unpublished observations). RT-PCR and sequencing of the *QPT* coding region in the *nic2-1* mutant strain reveal a single nucleotide deletion at nucleotide 559 that leads to a frameshift. The amino acid sequence changes at Gly_187_ and generates a premature stop codon at amino acid 240 (Figure 3A; Figure S3). The mutant protein contains all the conserved QA-binding sites (Figure S3, blue reversed triangles) but the α8-12 helices and β8-12 sheets required to form α/β barrel are missing [32]. Transformation with a BAC clone (38P5) containing a full-length *QPT* gene yields 8 independent 3-AP-resistant colonies.

The *Chlamydomonas NMNAT* gene was predicted to contain 4 exons and encode a 234 aa protein [15]. However, RT-PCR, nested PCR and DNA sequencing shows the coding region of *Chlamydomonas NMNAT* is composed of only 2 exons that encodes a 524 aa protein, as predicted by the GreenGenie2 algorithm [33]. Sequence alignment reveals that *Chlamydomonas* NMNAT contains extra glycine/proline/glutamine-rich sequences compared to NMNAT proteins from other organisms (Figure S4). The *Chlamydomonas NMNAT* gene is ∼262 kb away from the *IDA2* gene [34] and maps to LG XV [31], near where the *NIC1* gene maps [25]. Sequencing of the *NMNAT* genomic DNA from a *nic1-1* strain indicates two adjacent nucleotide changes A_1406_C_1407_→T_1406_T_1407_ result in Q_345_H, Q_346_stop (Figure 3A; Figure S4). These nucleotide changes are likely to generate a truncated NMNAT in the *nic1-1* mutant strain. The 3-AP-sensitive *nic1-1* mutant phenotype is leaky and reverts at a low frequency of ∼1 spontaneous 3-AP-resistant colony per 10^8^ cells. Therefore, a co-transformation approach was used for gene complementation. BAC DNA (10M24) or plasmid DNA (pNIC1-56) containing the full-length *NMNAT* gene was co-transformed with a paromomycin-resistant gene (*APHVIII*; [35]). A subset of the paromomycin-resistant colonies (5/20 for BAC and 2/2 for plasmid transformation) showed resistance to 3-AP.

The *Chlamydomonas NS* homolog maps between GP220 and GP441, which are two molecular markers on LG X [31], where *nic13-1* maps [25]. RT-PCR and DNA sequencing from wild-type cells indicate this gene contains 20 exons that encode an 832 aa protein. The intron between exons 2 and 3 is unusually long (∼3.5 kb) for a *Chlamydomonas* gene (Figure 3A). Similar to *Chlamydomonas* NMNAT, *Chlamydomonas* NS contains extra sequences not found in other organisms. This insert is rich in alanine residues (Figure S5). The coding region of *NS* in *nic13-1* was sequenced and a triple nucleotide substitution (TCC→ATT) is predicted to produce a S_740_I change (Figure 3A). The S_740_ is highly conserved among plants, green algae, and mammals (Figure S5). Further evidence that this point mutation is responsible for the mutant phenotype is provided by reversion analysis. We reasoned that a single base change of ATT (I) to AGT (S) would generate an I_740_S reversion. This change would also create a *Sfc*I site (TTCTAC**AG** **T**A), which is not present in either wild-type or *nic13-1* cells (Figure 3B). Eighteen 3-AP-resistant colonies were isolated following UV mutagenesis of *nic13-1* cells. Six of them contained a *Sfc*I site as monitored by PCR and restriction digestion of the product (Figure 3B). Of these, three revertants were randomly selected for sequencing. The ATT→AGT change was confirmed in all three selected revertants. The other 12 revertants were not analyzed. Transformation of *nic13-1* cells with BAC (10H24) produced two 3-AP-resistant colonies and this provides further evidence that the mutation in *NS* is responsible for the *nic13-1* mutant phenotype.

### Salvage biosynthesis of NAD^+^ in *Chlamydomonas* uses the pathway found in mammals

**Figure 4:**
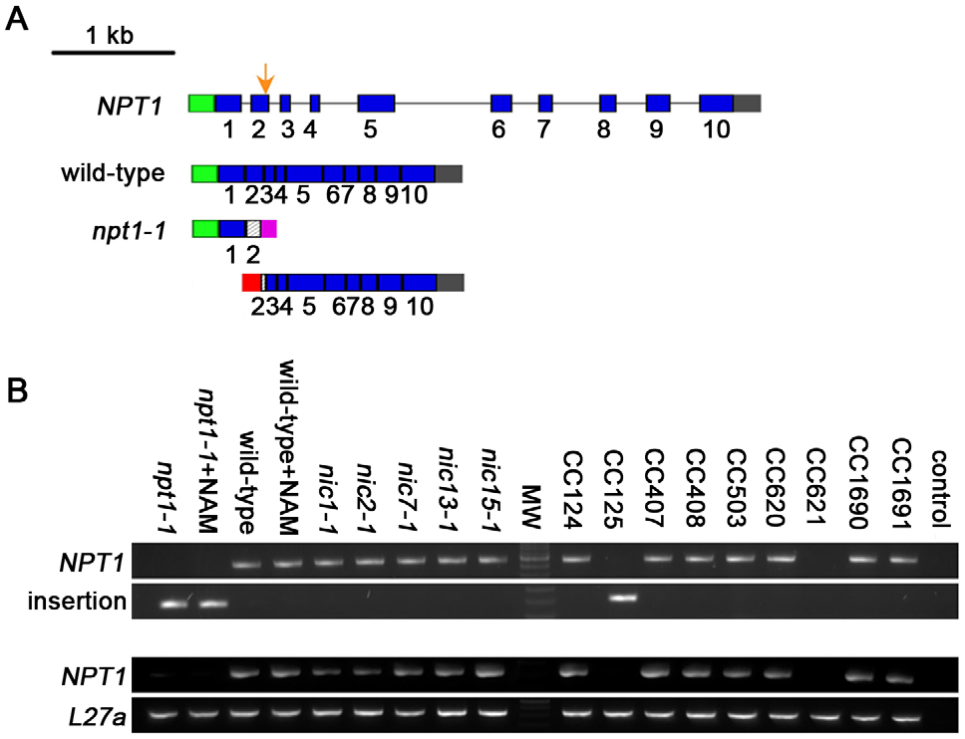
*Chlamydomonas* strain CC-125 contains an insertion in the *NAMPT* gene. (A) Schematic diagram shows gene structure of the *Chlamydomonas NAMPT* gene. An insertion in exon 2 in the *npt1-1* mutant strain is indicted by an orange arrow. Blue solid box, exon; black solid line, intron; green solid box, 5′ UTR; gray solid box, 3′ UTR; magenta and red solid boxes, sequences from the putative transposon(s); hatched boxes, incomplete regions of exon 2. (B) PCR amplification of *NPT1* genomic DNA around the insertional region. Upper panel, primers spanning the insertional point can amplify the wild-type *NPT1* but not *NPT1* with an insert. Lower panel, one primer binds to the inserted sequence while the other binds to *NPT1*. Thus only *NPT1* with the specific inserted sequence can be amplified. (C) RT-PCR amplification of the whole coding region of *NPT1*. *L27a*, a ribosomal protein gene serves as a control [84]. Control, no DNA template was added in PCR.

Since *Chlamydomonas nic* mutants can utilize both NAM and NMN (Figure 2), two metabolites found in the 2-step salvage pathway, we propose that *Chlamydomonas* uses this pathway to synthesize NAD^+^. The 2-step salvage pathway utilizes two enzymes, NAMPT and NMNAT. A NAMPT homolog, which is ∼30% identical to human NAMPT, was identified via sequence similarity search (Figure S6). RT-PCR and DNA sequencing indicated the *Chlamydomonas NAMPT* gene (*NPT1*, GenBank HM061641) contains 10 exons and encodes a 543 aa protein (Figure 4A) in CC-124 wild-type cells. However, we failed to amplify the full-length *NPT1* transcript (∼2.2 kb) from another wild-type strain (CC-125) (Figure 4C). Further investigation using 3′ RACE and 5′ RACE shows that an insertion in exon 2 of the *NPT1* gene is present in the CC-125 strain (Figure 4A, 4B). This region was partially sequenced and the inserted DNA sequence maps to multiple places in the genome and is likely to contain one or more transposable elements. The insertion causes two truncated *NPT1* transcripts in the CC-125 strain. The first one is ∼0.6 kb long and contains the endogenous *NPT1* promoter and ends within ∼100 bp of the inserted DNA (Figure 4A). It is predicted to contain an open reading frame (ORF), which encodes a 127 aa protein. This predicted protein contains the first 60 aa of the conserved phosphoribosyltransferase domain, which is ∼450 aa long. The second transcript is ∼1.8 kb long and starts with ∼130 bp of the inserted DNA (Figure 4A). This truncated transcript contains part of exon 2 and the rest of the gene and is predicted to contain an ORF encoding a 435 aa protein. The predicted protein lacks the first 65 aa of the conserved phosphoribosyltransferase domain. Thus, we conclude that the CC-125 strain carries a defective NPT1 and we name the allele *npt1-1* (nicotinamide phosphoribosyltransferase). All the *nic* mutants contain a full-length *NPT1* transcript (Figure 4B, 4C).

Given that CC-124 and CC-125 strains are considered to be “wild-type” strains in the *Chlamydomonas* community, we tested whether any other wild-type strains contain this insertion. The CC-124 and CC-125 strains originated from the 137c zygotic isolate by Smith in 1945 [36]. The meiotic progeny from 137c was distributed to Sager in 1953 and to Ebersold in 1955. Four of the strains we tested, CC-1690 (21gr), CC-1691 (6145c), CC-407 (C8), and CC-408 (C9), originated from the Sager 1953 branch. The other three strains, CC-503 (*cw9*2, used for the genomic sequence by JGI), CC-620 (R3), and CC-621 (NO), as well as CC-124 and CC-125, all came from the Ebersold branch. Genomic DNA PCR was used to identify the transposon insertion event while cDNA PCR indicated the presence/absence of the *NPT1* transcript. Most of the strains have an intact *NPT1* gene (CC-407, 408, 503, 620, 1690, and 1691). The CC-621 strain (upper panel, Figure 4B) has an insertion in exon 2 of *NPT1*, but the insertional sequence is not identical to the CC-125 insertion since the PCR primers that recognize the insertion in CC-125 failed in CC-621 (lower panel, Figure 4B). As expected, the CC-621 strain also does not express the full-length *NPT1* transcript (Figure 4C). In contrast to the *nic* mutant strains, the *npt1-1* mutant strains, CC-125 (*npt1-1*) and CC-621 (*npt1-2*), show no obvious growth defect on rich medium or medium supplied with 3-AP (Figure 2 and data not shown).

Sequence similarity searches indicate that *Chlamydomonas* does not contain four of the six enzymes required to synthesize NAD^+^ from the *de novo* tryptophan pathway and it is missing a homolog of NAPRT from the 4-step salvage pathway. *Chlamydomonas* has genes for the IDO and KMO enzymes in the tryptophan pathway and has a NAMase homolog in the 4-step salvage pathway. Given the apparent incompleteness of either of these two pathways, we hypothesized that *Chlamydomonas* is unable to synthesize NAD^+^ via the tryptophan pathway or the 4-step salvage pathway (Figure 1).

If the hypothesis that *Chlamydomonas* contains only the *de novo* aspartate pathway and the 2-step salvage pathway is correct, then double mutant strains containing the *npt1-1* mutation as well as one of the *nic* mutations should be lethal and fail to grow on medium supplied with NAM. Otherwise, if there were additional NAD^+^ synthesis pathways available, then *npt1-1*; *nic2-1* or *npt1-1*; *nic15-1* double mutants should survive on NAM medium. We performed crosses between the *nic* mutants and the *npt1-1* mutant strain. Genotypes of the meiotic progeny were scored based on their viability (*NIC*) or inviability (*nic*) on medium containing 3-AP, and the presence (*npt1*) or absence (*NPT1*) of the *NPT1* transposon insertion, which was tested by genomic DNA PCR. The results are summarized in Table 2. Out of 198 viable progeny generated from 4 different crosses, no *npt1*; *nic* double mutants were recovered on NAM medium. Because addition of NMN rescued the *nic* mutants and it is downstream of the NAMPT catalyzed step, we expected that *npt1*; *nic* double mutants should be viable on medium supplied with NMN. However, we found that no *npt1*; *nic* double mutants were isolated out of 148 viable progeny on NMN medium. One potential cause may be the hydrolysis or inefficient uptake of NMN by meiotic progeny compared to vegetative cells. Alternatively, the *NIC1* message may not be expressed in meiotic progeny (Figure 2). Thus, based on the results from these genetic crosses between the *nic* mutants and the *npt1-1* mutant, we conclude that *Chlamydomonas* synthesizes NAD^+^ via the *de novo* aspartate pathway and the 2-step salvage pathway and it is very unlikely that there is additional NAD^+^ biosynthesis pathway.

**Table 2:** Progeny analysis from crosses between *npt1-1* and *nic* mutant strains.

*Genotypes of meiotic progeny analyzed from npt1-1 x nic*	*nic1-1*	*nic2-1*	*nic13-1*	*nic15-1*
**R+NAM Medium**				
*NIC*; *NPT1*	6	27	8	27
*NIC*; *npt1*	16	26	6	26
*nic*; *NPT1*	12	20	8	16
*nic*; *npt1*	0	0	0	0
**R+NMN Medium**				
*NIC*; *NPT1*	15	19	1	15
*NIC*; *npt1*	7	18	5	24
*nic*; *NPT1*	9	16	2	17
*nic*; *npt1*	0	0	0	0

### Transcriptional regulation among genes involved in the NAD^+^ biosynthesis pathways

**Figure 5:**
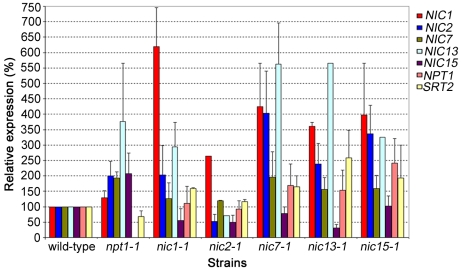
Gene expression in wild-type, *npt1-1*, and *nic* mutant cells. Relative real-time RT-PCR was used to detect transcripts. Results represent 2 biological replicates and standard errors are indicated by error bars.

Previous studies on bacterial and mammalian NAD^+^ biosynthesis indicate that transcriptional regulation among genes involved in the pathways is common. In *Escherichia coli* and *Salmonella enterica*, expression of *nadB* (encodes ASO) and *nadA* (encodes QS) is regulated by *nadR*, which has NMNAT activity [37], [38]. In mammals, the circadian expression of *NAMPT* is partially regulated by SIRT1, the enzyme that converts NAD^+^ to NAM, which is the substrate of NAMPT [39]. To investigate whether transcriptional regulation among the *NIC* genes and *NPT1* exists in *Chlamydomonas*, we measured transcript levels of these genes in *nic* and *npt1-1* mutants by quantitative real-time RT-PCR (qRT-PCR, Figure 5). Changes were not considered significant unless a gene is >2-fold upregulated or <2-fold downregulated when compared to its expression level in wild-type cells.

The first step in the *de novo* aspartate pathway, which is rate limiting in bacteria, is catalyzed by ASO, encoded by *NIC15*. As expected, mutations in the downstream enzymes (*nic1-1*, *nic2-1*, and *nic13-1*) result in reduced *NIC15* transcript while the *npt1-1* mutation causes a 2-fold elevation in *NIC15* transcript level. The *NIC7* transcript level was not affected in any of the mutants tested. *NIC2* and *NIC13* transcript levels are upregulated in the *npt1-1* mutant and in all the *nic* mutants except *nic2-1*. The *NIC1* transcript level is upregulated in the *nic* mutants but not in *npt1*. Finally, the expression level of *NPT1* is only upregulated in the *nic15-1* mutant strain.

Overall, gene expression of the *NIC*, *NPT1* and *SRT2* genes shows complicate patterns. No single gene is upregulated or downregulated in all mutants and no single mutant shows clear upregulation or downregulation of all genes involved in a pathway. This result suggests that in addition to regulation at the transcription level, NAD^+^ biosynthesis may be regulated post-transcriptionally.

### The link between NAD^+^ biosynthesis in *Chlamydomonas* and longevity

**Figure 6:**
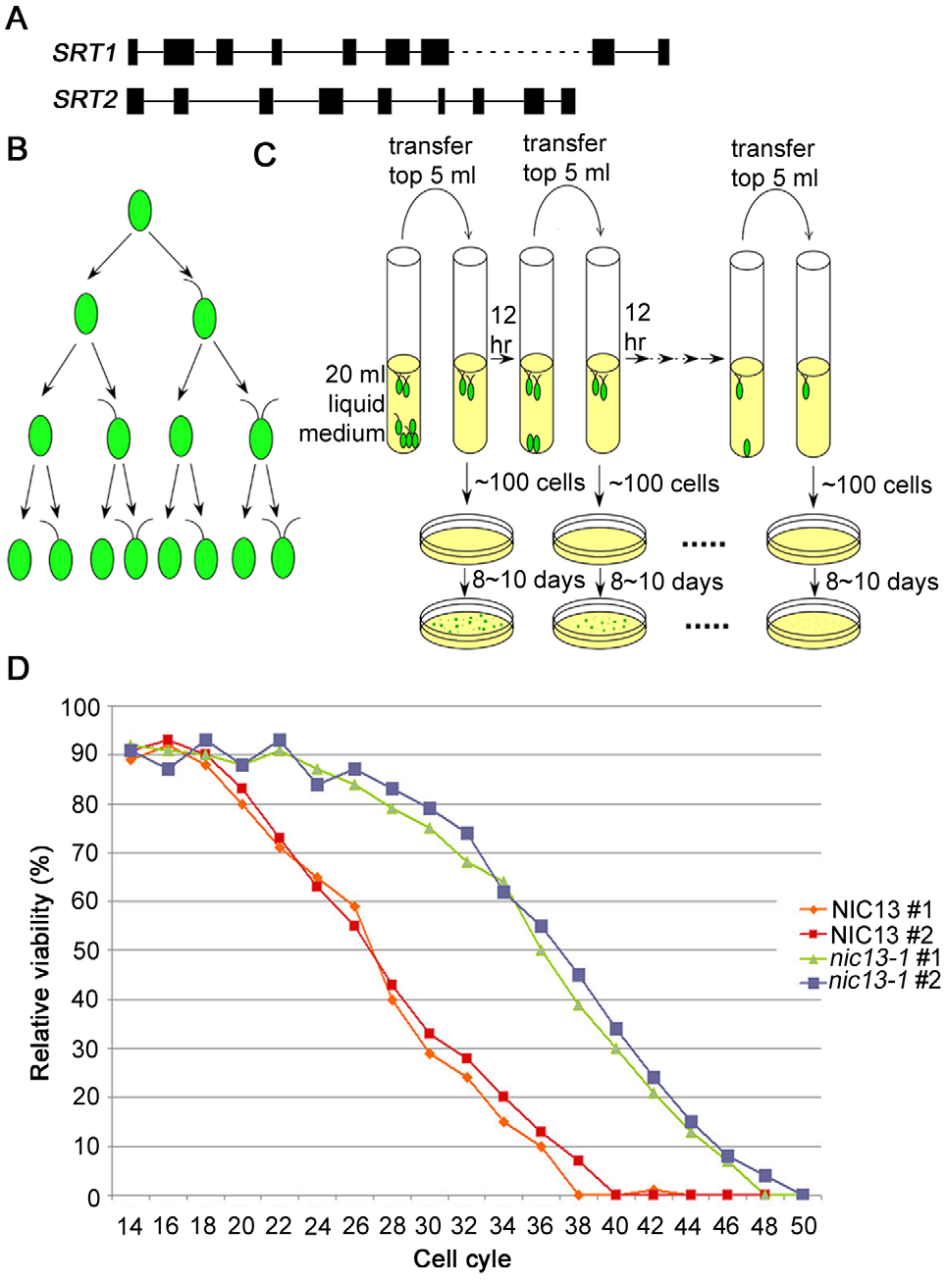
*Chlamydomonas SRT2* and life span extension in *Chlamydomonas nic13-1* mutant strain. (A) Schematic diagram shows gene structures of *Chlamydomonas SRT1* and *SRT2*. (B) Pedigree of the *uni3-1* mutant strain regarding flagellar numbers, redrawn from Dutcher and Trabuco [43]. (C) Schematic diagram shows how the aging experiment was performed with *Chlamydomonas* cells. (D) Life span in wild-type and *nic13-1* cells. Results from 2 biological replicates of each strain are shown.

In studies of yeast, worms, and mammals, upregulation of NAD^+^-dependent histone deacetylase Sir2/SIRT1 is correlated to longevity [40], [41]. In rice, RNA interference of *OsSRT1* leads to DNA fragmentation and programmed cell death [42]. We wanted to test if SIR2 homologs play a similar role in algae. A sequence similarity search using SIR or SIRT proteins finds two proteins in *Chlamydomonas*. We named the one most to similar to yeast Sir2 protein SRT2. RT-PCR and sequencing show that the *Chlamydomonas SRT2* gene contains 9 exons (Figure 6A) and encodes a 320 aa protein (GenBank HM061642). Sequence alignment (Figure S7) shows that this protein contains the NAD-dependent catalytic core domain and is closely related to human SIRT6, SIRT7, and plant SRT proteins, which are class IV SIRT proteins. The second *SIR2*-like gene, *SRT1*, is most similar to human SIRT4 [11], which is a mitochondrial protein. This gene encodes a 399 aa protein (GenBank HM161714) and belongs to the class II sirtuin family (Figure 6A and Figure S7).

Since Sir2-like proteins are involved in the enzymatic step of converting NAD^+^ to NAM, we tested the expression of *Chlamydomonas SRT1* and *SRT2* by qRT-PCR. The transcript level of *SRT1* is extremely low and we could not obtain informative qRT-PCR data. Thus, we focused on *SRT2* transcript levels in wild-type, *nic* and *npt1-1* mutants (Figure 5), and find that *SRT2* remained unchanged in all strains except in *nic13-1* cells, which show a ∼2.5 fold increase.

We then tested whether this increase of *SRT2* expression in *nic13-1* cells affects *Chlamydomonas* cell longevity. We took advantage of the *Chlamydomonas uni3-1* cells, which have a deletion of delta-tubulin. A pedigree analysis of this mutant suggested that the flagellar number is a metric of the mitotic age of cells (Figure 6B). As shown by Dutcher and Trabuco, biflagellate cells are only produced by *uni3-1* cells that have undergone at least two cell divisions [43]. Aflagellate cells never produce biflagellate daughters, but a uniflagellate or biflagellate *uni3-1* cell produces one aflagellate daughter cell and one biflagellate daughter cell. We suggest that the biflagellate cell is the equivalent of using the mother cell in budding yeast as a marker of generational age. Having two flagella allows a cell to swim effectively to the air-liquid interface of the medium, while an aflagellate or uniflagellate daughter cell sinks to the bottom of the culture tube. The biflagellate daughter cells can then be transferred to a new test tube and of the number of generations that the *uni3-1* cells undergoes can be monitored (Figure 6C). As illustrated in Figure 6D, *NIC13*; *uni3-1* biflagellate cells complete 38–40 cell cycles. On the other hand, *nic13*; *uni3-1* cells complete 48–50 cell cycles. This represents a ∼25% increase in reproductive capacity. We assayed a *nic2-1*; *uni3-1* strain since it does not have increased *SRT2* levels but has a synthesis defect and found that it completed 37 cell cycles like wild-type cells (data not shown). Therefore, we conclude that NAD^+^ biosynthesis in *Chlamydomonas* can affect life span and this might be achieved by alternating the expression level of *Chlamydomonas SRT2*.

## Discussion

The essential roles of NAD^+^ in many metabolic oxidation-reduction reactions are well established. Recent studies link its function to transcriptional regulation [44], epigenetic regulation [45], longevity [2], cell death [46], neurogeneration [47], circadian clocks [48], and signal transduction [49]. Understanding its biosynthetic pathways will facilitate understanding of lifespan extension [50], disease regulation [51], drug design [52], as well as evolution [53].

Recent studies on NAD^+^ biosynthesis indicate that pathways in different organisms are more diverse than expected. The tryptophan pathway, which was thought to be eukaryotic specific, was identified in several bacteria [54]. The aspartate pathway, which was considered only prokaryotic, is present and essential to *Arabidopsis thaliana* [28]. An organism may contain all the enzymes required for more than one pathway, but a single pathway is predominantly used. *Bacillus subtilis* can synthesize NAD^+^ via aspartate or the 4-step pathway but only genes involved in the conversion from NA to NAD^+^ are indispensable [55]. *Arabidopsis thaliana* contains the aspartate pathway and the 4-step pathway. However homozygous *ASO* and *QS* mutants, which specifically affect the aspartate pathway, are inviable [28]. In mammals, the enzyme NAMPT, which is the rate-limiting enzyme in the 2-step pathway, is essential even though organisms harbor all the enzymes required to synthesize NAD^+^ from tryptophan [56]. However in *D. melanogaster* and *C. elegans*, there is only one pathway. The *de novo* NAD^+^ synthesis pathway is incomplete and they rely on the NAMase-dependent salvage pathway to synthesize NAD^+^ [53].

Our study indicates that *Chlamydomonas* can synthesize NAD^+^ via the aspartate pathway, which is found in land plants and bacteria, or the 2-step salvage pathway, which is found in mammals. This combination in *Chlamydomonas* makes it a great model for the study of NAD^+^ biosynthesis. Similar to *Arabidopsis*, *Chlamydomonas* contains one copy of each gene that encodes the *de novo* pathway enzymes and the *Chlamydomonas* proteins are 51%∼63% identical to *Arabidopsis* homologs. However, unlike *Arabidopsis* mutants, which are lethal when homozygous [28], [57], the *Chlamydomonas nic* mutants show conditional lethality as they are rescued by the addition of NAM or NMN to the medium. Thus, the effects of loss of function mutations, which can not be studied in *Arabidopsis*, can be easily analyzed in *Chlamydomonas*. In mammals, NAMPT is essential. It is encoded by three different genes and the proteins localize to different cellular compartments. In addition, mammal NAMPT has an extracellular form; both intracellular (iNAMPT) and extracellular (eNAMPT) forms are involved in NAD^+^ synthesis and in regulation of insulin secretion in pancreatic β cells [58]. *Chlamydomonas* contains only one copy of NAMPT (*NPT1*). The *npt1-1* mutant has no growth defect but none of the *nic*; *npt1-1*double mutants are viable (Table 2). Since *Chlamydomonas* cells are haploid and easy to maintain, this mutant provides an alternative for screening for NAMPT-blocking drugs. The potential drugs would have no effect on wild-type cells but would be lethal to *nic* cells.

In mammals 3-AP acts as an analog of nicotinic acid and inhibits the 4-step salvage pathway. In *Chlamydomonas*, 3-AP prevents the rescue of *nic* mutants by NMN and greatly suppresses the rescue by NAM. The easiest explanation for these results would be that *Chlamydomonas* has the 4-step salvage pathway and it is active. However, the *Chlamydomonas* genome has only three of the four enzymes; the genome assembly is missing the key enzyme, NAPRT. It remains a possibility that *Chlamydomonas* has a *NAPRT* gene, but it is not present in the assembled genome sequence. Two lines of evidence suggest that a functional NAPRT is not likely to be present in *Chlamydomonas*. First, using 40 million Illumina transcriptome reads (1.2 Gb of sequence) from three independent mRNA preparations, we find evidence for transcription of the first 45 amino acids of NAPRT using a splice aware assembly algorithm, but find no evidence for the transcription of the rest of the gene that contains all of the known active sites needed for function [59], [60] (unpublished data). Given the high identity of this protein from microalgae to mammals, it is likely either that the rest of the *Chlamydomonas NAPRT* gene was lost or the gene is not transcribed beyond the first 135 bps of the open reading frame. Second, the genome sequence of *Volvox carteri* (http://genome.jgi-psf.org/Volca1/Volca1.home.html), a multicellular green alga that shared a common ancestor with *Chlamydomonas* around 35 million years ago [61], also lacks *NAPRT*. Therefore, we suggest that *Chlamydomonas* cells do not have a functional copy of NAPRT. It remains an open possibility whether an alternative enzyme without sequence similarity exists in *Chlamydomonas*.

**Figure 7:**
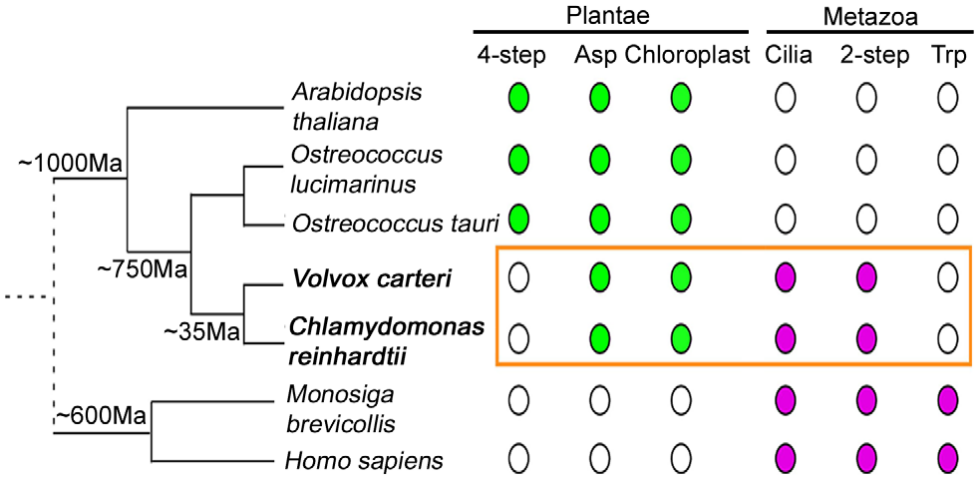
NAD^+^ biosynthesis in *Chlamydomonas* reveals evolutionary aspects. Evolutionary distances between different organisms are indicated on the left. Branch lengths are not to scale. *Chlamydomonas reinhardtii* and *Volvox carteri* are indicated in bold letters. Filled dots indicate the presence of a trait in organisms and open dots indicate absence. Traits found in photosynthetic organisms are indicated in green and traits associated primarily with the animal lineage are indicated in magenta. The distinct traits of NAD^+^ biosynthesis in *Chlamydomonas* and *Volvox* are highlighted in the orange box. 2-step, 2-step salvage pathway of NAD^+^ biosynthesis; 4-step, 4-step salvage pathway of NAD^+^ biosynthesis; Asp, *de novo* biosynthesis of NAD^+^ from aspartic acid; Trp, *de novo* biosynthesis of NAD^+^ from tryptophan.

Our study on *Chlamydomonas* also provides important insights into the evolution of NAD^+^ biosynthesis (Figure 7). Through sequence similarity searches, we find that *Volvox* contains enzymes required for the aspartate *de novo* pathway and the 2-step salvage pathway. Given the common ancestor, it is not surprising that both of them contain NAMPT, the enzyme that is unique to the 2-step pathway. Two unicellular green microalgae, *Ostreococcus lucimarinus* and *Ostreococcus tauri* [62], [63], contain enzymes required for the aspartate *de novo* pathway and the 4-step salvage pathway. *Ostreococcus* are believed to have diverged from *Chlamydomonas* around 750 million years ago, ∼250 million years after the separation of chlorophytes (green algae) and streptophytes (seed plants) [64]. The unicellular choanoflagellate *Monosiga brevicollis*, which is considered the progenitor to animals and separated from other metazoans more than 600 million years ago [65], has enzymes found in the tryptophan pathway and the 2-step pathway, as in animals. *Chlamydomonas*, which has remnants of four pathways, may suggest that an ancestral organism had multiple pathways and that most organisms have retained only a subset.

In *Arabidopsis*, the first three enzymes, ASO, QS, and QPT, are localized to chloroplasts. It is currently unclear where the other two proteins, NMNAT and QS, are localized. When we used Predotar [66] and TargetP [67] for *Chlamydomonas* protein localization prediction, ASO, QS, and NS are predicted to localize to mitochondria by both programs. NMNAT is predicted to be in the mitochondria by TargetP while Predotar gives no specific location. The localization of QPT is unspecified by either program. The actual localization of *Chlamydomonas* proteins will require experimental determination. If all *Arabidopsis* enzymes are localized to chloroplasts while all *Chlamydomonas* enzymes are not, it would suggest that having NAD^+^ biosynthesis in plastids happened after the separation of green algae and seed plants.

As illustrated in Figure 1, NMNAT is an essential enzyme utilized by all NAD^+^ biosynthetic pathways. We observed that *NIC1* has a low basal expression level in wild-type cells compared to the other *NIC* genes, but is upregulated 2∼6 fold in various *nic* mutants. This upregulation is consistent with the hypothesis that NMNAT is the key enzyme involved in all NAD^+^ biosynthetic pathways and any mutation along the pathway affects the expression of *NIC1* significantly. The nonsense mutation found in *nic1-1* cells presumably generates a truncated protein that must be partially functional as we would expect that a null mutant would disrupt both pathways and be lethal like the double *nic*; *npt1-1* mutant strains. The truncated protein has the catalytic motif residue H30 but only one of two substrate binding motif residues (W98 and not R224) [68], [69].

Through our sequence similarity search, only two of the six homologs in the *de novo* synthesis pathway starting from tryptophan were identified. Previously, *nic1-1* and *nic4* mutants were reported to grow on medium supplemented with 3-HA, a metabolite produced in the tryptophan pathway [18]. We find that the growth defect of *nic1-1* cannot be rescued by the addition of 3-HA (Figure 1) and this agrees with our finding that *NIC1* encodes NMNAT, which acts downstream of 3-HA. In addition, 3-HAO, the enzyme that uses 3-HA as a substrate, is not present in the *Chlamydomonas* genome sequence. The *nic4* mutant is no longer existent in the *Chlamydomonas* strain collection and was never mapped ([19], www.chlamydb.html). Therefore, we are unable to test its grow ability on medium provided with 3-HA. Similar to our finding, 3-HA and other intermediates found in the tryptophan pathway fail to rescue nicotinamide requiring mutants in *Chlamydomonas eugametos* [70]. Recent studies indicate that nicotinamide riboside (NR) and nicotinic acid riboside (NaR) are NAD^+^ precursors in yeast and mammalian cells [71]–[73]. Enzymes involved in the NR and NaR salvage pathways include nicotinamide riboside kinase (NRK1), purine nucleoside phsophorylase (PNP1), uridine hydrolase (URH1), and methylthioadenosine phosphorylase (MEU1). Similarity searches using yeast protein sequences identified only one PNP1-like protein in *Chlamydomonas*, but none of the other proteins. Thus, it is unlikely that *Chlamydomonas* contains the NR/NaR salvage pathway.

In the study of longevity, several model organisms (*S. cerviseae*, *C. elegans*, *D. melanogaster*, and mouse) have been widely used. Caloric restriction leads to extended life span in these organisms, but the mechanisms behind these findings are not well understood. Studies indicate that caloric restriction-mediated longevity links to upregulation of Sir2 in yeast [41], [74], flies [75], and mammals [76] but is independent of Sir2 expression in worms [77]. However, increasing the dosage of *SIR2* in *C. elegans* leads to longer life span [40]. Our observation that mutant cells with a longer life span have increased *SRT2* expression suggests a link between *Chlamydomonas SIR2*-like genes and longevity. It is intriguing that only the *nic13-1* mutant strain has increased levels of *SRT2*. Since *nic13-1* mutants should have increased levels of the intermediate, NaAD, we attempted to ask if exogenous NaAD altered *SRT2* levels. Exogenous NaAD failed to rescue upstream mutants, which suggests that it was not effectively imported into cells (Figure 2).

We assayed replicative aging in *Chlamydomonas* using centriole or basal body age as our marker. In the *uni3-1* populations, the biflagellate cells contain a grandmother centriole (at least three cell cycles old) and a daughter centriole. We can recover biflagellate cells by virtue of their ability to swim. The cells that are biflagellate represent the oldest cells in the population. We find that wild-type cells fail to divide after 38–40 generations while the *nic13-1* mutant continues for at least 10 more cell divisions. We suggest that this aging may include aging of the centrioles. Recent studies on fruit fly germline stem cells [78] and mouse neural progenitor cells [79] indicate that the mother centriole stays with the self-renewing daughter stem cell while the daughter centriole goes with the differentiating daughter cell. As cells age, misorientation of centrioles accumulates and eventually causes cell cycle delay or arrest in mouse neural progenitor cells. Using *Chlamydomonas* as a model system to study aging, we can further pursue the link between NAD^+^ metabolism, Sir2-like genes, and centriole aging. Whether overexpression of *SIR2* in *Chlamydomonas* causes extended life span as shown in other organisms needs additional experimentation.

A recent study on mammalian SIRT1 indicates that it is involved in regulation of circadian rhythm via transcriptional regulation of several key genes [80]. It is currently unclear whether other SIRT proteins have similar effect on circadian rhythm. Given that synchronous *Chlamydomonas* cell culture can be easily achieved by alternating light/dark cycles, we foresee *Chlamydomonas* as a model to explore the effect of *SRT2* (SIRT6-like) and *SRT1* (SIRT4-like) on circadian rhythms [81].

In conclusion, the results presented in this work underscore several key advantages of using *Chlamydomonas* as a model system for further studies of NAD^+^ metabolism. The *Chlamydomonas* genome contains a single copy of each of the proteins that make up the plant-specific *de novo* NAD^+^ biosynthesis pathway. However, unlike *Arabidopsis*, which is homozygous lethal for the first three enzymes, all five *Chlamydomonas* mutants show conditional lethality. Consequently, *Chlamydomonas* will facilitate future studies on metabolites involved in NAD^+^ biosynthesis. *Chlamydomonas* also contains a single copy of the genes in the mammal-specific 2-step NAD^+^ salvage pathway. The fact that mammals contain multiple isoforms of NAMPT and that this enzyme is essential to viability impede NAMPT-blocking drug studies in mammal-based model systems. As such, NAMPT targeted drug screens using *Chlamydomonas* avoid the many confounding factors that are inherent in current screening methods. Our centriole aging results demonstrate how *Chlamydomonas* may be a valuable model organism for future studies in cellular and organelle aging.

## Materials and Methods

### *Chlamydomonas* strains and spotting assay

*Chlamydomonas reinhardtii* strains, CC-14 (*nic15-1*; *mt+*), CC-124 (mt−), CC-125 (mt+), CC-407 (C8, mt+), CC-408 (C9, mt−), CC-503 (*cw92*; mt+), CC-599 (*nic1-1*; *mt+*), CC-620 (R3, mt+), CC-621 (NO, mt−), CC-864 (*nic13-1*; *mt+*), CC-1079 (*ac12*; *thi9*; *nic2-1*; *mt+*), CC-1690 (21gr, mt+), CC-1691 (6145c, mt−), and CC-3657 (*nic2-1*; *mt+*), were obtained from *Chlamydomonas* Center (Duke University) and maintained on solid rich growth (R) medium [82] or medium containing 2 µg/ml (16 µM) nicotinamide (NAM). To confirm that the *nic* mutant strains show the Nic^−^ phenotype, cells were plated on R medium containing 15 µl/l (16.5 mg/l) 3-acetylpyridine (3-AP) [20]. The original *nic15-1* strain acquired from the *Chlamydomonas* Center failed to confer sensitivity to 3-AP, which suggests the possibility of a revertant or an extragenic suppressor. A backcross to the wild-type strain produced progeny sensitive to 3-AP, which reveals the presence of an extragenic suppressor in the original stock culture. The 3-AP sensitivity phenotype of the *nic2* strain (CC-3657) was difficult to score, so a second strain CC-1079 (*ac12*; *thi9*; *nic2-1*; *mt+*) was backcrossed to wild-type cells several times to generate an *AC12*; *THI9*; *nic2-1* strain that confers sensitivity to 3-AP. For the spotting assay, 10^4^ cells were spotted on R medium or R+3-AP medium supplemented with one of the following compounds: 10 µM NAM, 10 µM nicotinamide mononucleotide (NMN, dissolved in water), 10 µM nicotinate adenine dinucleotide (NaAD, dissolved in water), 10 µM nicotinic acid (NA, dissolved in water), or 10 µM 3-hydroxyanthranilate (3-HA, dissolved in DMSO). The plates were placed under constant light at room temperature for 3 days before pictures were taken. All the reagents were obtained from Sigma (St. Louis, MO).

We have changed the linkage group names to chromosome names as specified in [15]. Linkage groups I-XI correspond to chromosomes 1–11. Linkage group XII/XIII is chromosome 12, and Linkage group XV is chromosome 14.

### Identification of *Chlamydomonas* homologs via sequence similarity search

Protein sequences of *Arabidopsis thaliana* ASO, QS, QPT, NMNAT, NS (listed in Table 1), human NAMPT (NP_005737), yeast SIR2 (NP_010242), and human SIRT4 (NP_036372) were used in TBLASTN against JGI (Joint Genome Institute) *Chlamydomonas reinhardtii* genome version 4.0 (JGI v4.0, http://genome.jgi-psf.org/Chlre4/Chlre4.home.html) with expected E-values less than or equal to 1E-5 (1E-3 for SIR2 and SIRT4). The resultant genes were checked for EST coverage. Genes without full-length EST coverage, *QS*, *NMNAT*, *NS*, *NAMPT*, *SRT1*, and *SRT2*, were subjected to exon-intron predictions using GreenGenie 2 (http://bifrost.wustl.edu/cgi-bin/greengenie2/greenGenie2) [33]. The predicted coding regions were used as guidelines in primer design for RT-PCR to amplify the actual coding regions of these genes.

### Colorfy for protein sequence alignment

Multiple sequence alignments (MSA) were color-coded using the online MSA column percentage composition coloring tool, Colorfy (http://bifrost.wustl.edu/colorfy). Colorfy takes as input any standard ALN format MSA (e.g. default CLUSTAL output) [83] and outputs the corresponding color-coded MSA.

### Amplification of coding regions by RT-PCR

*Chlamydomonas* total RNA was prepared as previously described [84]. Five µg of total RNA from wild-type cells were used for cDNA synthesis using a 3′ RACE poly (dT)-adaptor primer (Integrated DNA Technologies, Iowa City, IA) in a 50 µl reaction, which contains 1× RT buffer (Invitrogen, San Diego, CA), 10 mM DTT, 0.5 mM dNTP, 0.2 µM primer, 40 U of RNaseOUT (Invitrogen), and 200 U of SuperScript II reverse transcriptase (Invitrogen). The reaction was performed according to manufacturer's recommendation (Invitrogen). To remove RNA from the reaction, 2 units of RNase H (Invitrogen) were added at the end of reaction and incubated at 37°C for 20 min.

Amplification of the *NMNAT* coding region requires nested PCR due to highly repetitive sequences found in the gene. Five µl cDNA (1/10 of the reaction volume) from above was used in a 50 µl PCR reaction using a 3′ RACE primer and a gene-specific primer (nic1-3) that binds 4 nucleotides downstream of the predicted start codon. The reaction, which contained 1× KlentaqLA buffer (pH 9.2), 0.8 mM dNTP, 10% DMSO, 1 mM MgCl2, 0.5 µl KlentaqLA polymerase [85], was transferred directly from ice to a thermocycler (Bio-Rad, Hercules, CA) that was preheated to 93°C. The reaction conditions were: 93°C 5 min, 30 cycles of (93°C 15 sec, 53°C 15 sec, and 68°C 5 min), and 70°C 10 min. The resultant 2.2 kb fragment was used as template for a second round of amplification. A forward primer (nic1-20) that starts 98 nucleotides downstream of the predicted start codon and a reverse primer (nic1-24) that ends at the predicted stop codon were used. The resultant fragment was gel purified and subjected to DNA sequencing.

For amplification of other genes, 1 µl cDNA was used in a 20 µl PCR reaction containing 0.4 U Phusion DNA polymerase (Finnzymes, Woburn, MA), 1× GC buffer (Finnzymes), 0.2 mM dNTP, 3% DMSO, and 0.2 µM each of forward and reverse primers. The general reaction condition was 98°C 30 sec, 30 cycles of (98°C 10 sec, T°C 20 sec, and 72°C 30∼45 sec), and 72°C 10 min. T is the lower Tm of the primers calculated by Finnzymes' Tm calculator. Different sets of primers were used to cover the whole coding region of individual genes. The PCR products were subjected to gel purification and DNA sequencing to identify exon-intron boundaries.

### *Chlamydomonas* genomic DNA preparation

A DNA mini-prep protocol was modified [86] and used. Approximately 1×10^6^ cells were resuspended in 0.5 ml 1× TEN (150 mM NaCl, 10 mM EDTA pH 8.0, 10 mM Tris-HCl pH 8.0) and pipetted repeatedly until well resuspended. Cells were collected by centrifugation at 13,200 rpm for 10 sec in a microcentrifuge (Hermle Z233 M-2, Labnet, Woodbridge, NJ) and the supernatant was discarded. Cells were resuspended with 150 µl chilled water, followed by the addition of 300 µl SDS-EB buffer (2% SDS, 100 mM Tris-HCl pH 8.0, 400 mM NaCl, 40 mM EDTA pH8.0). DNA was extracted once with 350 µl phenol/chloroform (1∶1), followed by a second extraction using 350 µl chloroform. The volume was determined and twice the volume of 100% ethanol was added to precipitate DNA on ice for 30 min. Precipitated DNA was collected by centrifugation at room temperature for 10 min followed by a wash using 70% ethanol. DNA was dried using Savant SpeedVac (Thermo Scientific, Waltham, MA) and resuspended in 50 µl water. The concentration of DNA was determined by spectrophotometry at 260 nm (Eppendorf Biophotometer 6131, Westbury, NY). Approximately 20 ng of genomic DNA was used in PCR and the resultant PCR products were gel-purified and subjected to DNA sequencing. In the *nic1-1* cells, the region that carries mutations were amplified by the primer set nic1-10 and nic1-11. In the *nic15-1* cells, the region that contains a point mutation was amplified by nic15-3F and nic15-3R.

### BAC and plasmid DNA manipulation

*Chlamydomonas* BAC DNA was prepared using Qiagen Plasmid Midiprep kit. To prepare the pNIC15a plasmid, the BAC (32L22) DNA was digested with *Xma*I and a 6.1 kb fragment was isolated and cloned into a pBlueScript II SK vector (Stratagene, La Jolla, CA). This fragment contains a 1 kb upstream sequence, the full-length *NIC15* gene, and a 2.5 kb downstream sequence, which is predicted to be part of an unknown zinc finger protein (protein id 150664). To prepare the pNIC1-56 plasmid, a 7.1 kb *Kpn*I fragment from the BAC (10M24) DNA was cloned into a pBlueScript II SK vector. The plasmid contains a 0.7 kb upstream sequence, the full-length *NIC1* gene, and a 4.7 kb downstream sequence, which is predicted to contain an unknown protein that has a HAD-superfamily hydrolase domain.

### *Chlamydomonas* transformation

This protocol is modified from Iomini *et al* [87]. *Chlamydomonas* cells were inoculated in 100 ml liquid R medium for three days under continuous illumination with gentle shaking until cells reached a concentration of ∼5×10^6^ cells/ml. Cells were collected by centrifugation and treated with autolysin for 0.5 hr at room temperature to remove cell walls [19]. Autolysin-treated cells were chilled on ice for 10 min before collected by centrifugation at 4°C. Cells were gently resuspended on ice in R+100 mM mannitol to the final concentration of ∼4×10^8^ cells/ml. Two hundred fifty µl of cells (∼1×10^8^ cells) were used for transformation with 1 µg of BAC DNA or plasmid DNA with (the *nic1-1* strain) or without (the *nic15-1*, *nic2-1*, and *nic13-1* strains) the addition of 1 µg of pSI103, which confers resistance to paromomycin [35], for cotransformation. Cells and DNA were added to an electroporation cuvette (4mm gap, Bio-Rad) and incubated in a 16°C water bath for 5 min before electroporation, which was performed in a Bio-Rad Gene Pulser II with the following setting: 0.75 kv, 25 µF, and 50 Ω. Cells were electroporated with one pulse and incubated at room temperature for 10 min before transferring to 50 ml R+100 mM mannitol liquid medium and incubated overnight at room temperature with continuous illumination. Cells were resuspended gently in 1 ml 25% cornstarch in R medium and spread onto 5 R plates with 15 µl/l 3-AP (*nic15-1*, *nic2-1*, and *nic13-1* cells) or 5 R plates with 10 µg/ml paromomycin (*nic1-1* cells). Colonies appear within 5∼7 days at 25°C. The *nic1-1* transformants were tested subsequently on medium with 3-AP.

### UV-mutagenesis of the *nic13-1* cells

*nic13-1* cells were inoculated in 200 ml liquid R medium provided with 16 µM NAM for 4 days until cells reached a density of ∼10^6^ cells/ml. These cells were collected and spread evenly on an R+NAM medium plate. The cells were subjected to UV irradiation at 70 mJoules (Stratagene UV stratalinker 1800, Cedar Creek, TX) and recovered in the dark overnight. The plate was divided into 13 sections and cells were scraped off the plate and spread on 13 R+3-AP plates. 3-AP resistant colonies were observed one week later. Genomic DNA from individual cell lines, wild-type, and *nic13-1* cells were prepared as above and a short region was amplified by primers nic13-20F and nic13-3R by Phusion DNA polymerase. The PCR products were subjected to overnight digestion with *Sfc*I at 25°C and separated on a 2% agarose gel.

### Real-time RT-PCR

*Chlamydomonas* total RNA was extracted from ∼10^8^ cells using Qiagen RNeasy Mini Kit (Qiagen, Valencia, CA). Cells were homogenized by passing through a 20-gauge needle fitted to a 1 ml RNase-free syringe 20 times. The lysate was centrifuged and RNA extraction was performed according to manufacturer's recommendation. One microgram of total RNA from each strain was treated with 1 U of RNase-free DNAse I (Fermentas, Glen Burnie, MD) at 37°C for 30 min and the reaction was terminated by adding 1 µl of 25 mM EDTA and incubate at 65°C for 10 min. The DNAse I-treated RNA was added into a 20 µl reverse transcription reaction that contains 200 ng random primers (Invitrogen), 1× RT buffer (Invitrogen), 5 mM DTT, 0.5 mM dNTP, 20 U of RNaseOUT (Invitrogen), and 100 U of SuperScript III reverse transcriptase (Invitrogen). The reaction was performed according to manufacturer's recommendation (Invitrogen).

For real-time PCR, cDNA obtained from above was diluted 1∶10 and 2 µl was used in a 20 µl SYBR Green-PCR reaction [88] which contains 1× homemade PCR buffer (10 mM Tris-HCl, pH8.8, 50 mM KCl, 2 mM MgCl2; 0.1% Triton X-100); 1× SYBR Green I mix (1× SYBR Green, Molecular Probes; 10 nM Fluorescein, Bio-Rad; 0.1% Tween-20; 0.1 mg/ml BSA; 5% DMSO); 200 µM dNTP; 0.5 µM primers; and 1.6 µl TAQ DNA polymerase [89]. The reactions were carried out using a Bio-Rad iCycler iQ under the following conditions: 95°C 3 min, 40 cycles of (95°C 10 sec and 57°C 45 sec), followed by the melting curve program. The transcript levels of individual genes were standardized by an internal control, *CRY1*, which encodes the ribosomal protein S14 [90]. Gene expression was set to 100% in wild-type cells and the relative expression levels in various mutants were plotted as % increasing or decreasing related to transcript levels in wild-type cells. Results represent data from 2 biological replicates.

### *Chlamydomonas* aging

*nic13-1* cells were crossed to *uni3-1* (CC-4179) cells and the *nic13*-1; *uni3-1* double mutants were identified by 3-AP sensitivity and the presence of cells with 0, 1, or 2 flagella. Both *NIC13*; *uni3-1* and *nic13-1*; *uni3-1* cells were inoculated in 20 ml liquid R medium supplied with 16 µM NAM. The top 5 ml of liquid was transferred to a new test tube containing R+NAM every 12 hours. ∼100 cells were plated on R+NAM plates and the fraction of cells that formed colonies was counted under dissecting microscope after 8–10 days.

## Supporting Information

Figure S1 The *nic15-1* mutant strain has a missense mutation in aspartate oxidase (ASO). Protein sequence alignment of ASO from various organisms was performed by ClustalW [83] and the result is shown using Colorfy. Colorfy groups the twenty amino acids into eight separate conservation groups ({G, A}, {V, L, I}, {F, Y, W}, {C, M}, {K, R, H}, {D, E, N, Q}, {S, T}, {P}). Percentage composition is defined on a per column basis and categorized as Majority Identity, Conserved Minority or Insufficient Conservation. A column is Majority Identity when at least 61% of the amino acids in that column are identical. A column is Conserved Minority when at least 61% of the amino acids in that column belong to the same conservation group and no amino acid makes up more than 60% of that column. A column is Insufficient Conservation when its composition fails to satisfy any of the prior two conditions. Columns are colored based on percentage composition (Blue: 61 to 70; Green: 71 to 80; Gold: 81–90; Red: 91 to 100). Colors codes are divided into two shades, dark and light. A Majority Identity column can have up to two colors in the column: dark to indicate the positions of the identity amino acid and light to indicate positions of amino acids belonging to the same group as the identity amino acid. A Conserved Minority is colored the light color of the corresponding percentage composed of the majority amino acid group. Columns categorized as Insufficient Conservation are left uncolored. If a column satisfies Majority Identity at a lower percentage and Conserved Minority at a higher percentage, the Majority Identity categorization takes precedence and the column is colored per the Majority Identity percentage. The nucleotide sequences and the corresponding protein sequences around the mutation point for wild-type and *nic15-1* are shown in the box. The mutated nucleotide is underlined and the changed amino acid is shown in bold. The color of individual amino acids corresponds to their identity percentages among different organisms. At, *Arabidopsis thaliana*; Bs, *Bacillus subtilis*; Cr, *Chlamydomonas reinhardtii*; Ec, *Escherichia coli*; Ol, *Ostreococcus lucimarinus*; Os, *Oryza sativa*; Ot, *Ostreococcus tauri*; Pp, *Physcomitrella patens*; Pt, *Populus trichocarpa*; Vc, *Volvox carteri* ; Zm, *Zea mays*.
(2.46 MB TIF)

Figure S2 The *nic7-1* mutant strain has a missense mutation in quinolinate synthetase (QS). Protein sequence alignment of QS from various organisms was performed by ClustalW and the result is shown by Colorfy. The nucleotide sequences and the corresponding protein sequences around the mutation point for wild-type and *nic7-1* are shown in the box. The mutated nucleotide is underlined and the changed amino acid is shown in bold. The color of individual amino acids corresponds to their identity percentages among different organisms.
(2.26 MB TIF)

Figure S3 The *nic2-1* mutant strain has a deletion of a single nucleotide in quinolinate phosphoribosyltransferase (QPT). Protein sequence alignment of QPT from various organisms was performed by ClustalW and the result is shown by Colorfy. The conserved quinolinate-binding sites are indicated by blue reverse triangles. Partial nucleotide and the corresponding protein sequences for wild-type and *nic2-1* are indicated in the box. The deleted nucleotide is underlined in the wild-type. The deletion causes a frame shift that results in a stop codon (*) at amino acid 240. An, *Aspergillus nidulans*; Hs, *Homo sapiens*; Mm, *Mus musculus*; Nc, *Neurospora crassa*; Sc, *Saccharomyces cerevisiae*; Xl, *Xenopus laevis*.
(2.13 MB TIF)

Figure S4 The *nic1-1* mutant strain contains a premature stop codon in nicotinamide/nicotinate mononucleotide adenylyltransferase (NMNAT). Protein sequence alignment of NMNAT from various organisms was performed by ClustalW and the result is shown by Colorfy. Partial nucleotide and the corresponding protein sequences for wild-type and *nic1-1* are indicated in the box. The mutated nucleotides are underlined, and gray boxes indicate the codons. The amino acid changes are indicated by bold letters. The asterisk indicates a stop codon. Ce, *Caenorhabditis elegans*; Dm, *Drosophila melanogaster*; Sp, *Schizosaccharomyces pombe*.
(2.50 MB TIF)

Figure S5 The *nic13-1* mutant has a missense mutation in NAD^+^ synthase (NS). Protein sequence alignment of NS from various organisms was performed by ClustalW and the result is shown by Colorfy. Partial nucleotide and the corresponding protein sequences for wild-type and *nic13-1* are indicated in the box. The mutated nucleotides are underlined and the mutated amino acid is indicated by bold letters.
(4.63 MB TIF)

Figure S6 Sequence alignment of nicotinamide phosphoribosyltransferase (NAMPT) from various organisms. Protein sequence alignment of NAMPT was performed by ClustalW and the result is shown by Colorfy.
(1.93 MB TIF)

Figure S7 Sequence alignment of SIRT/Sir2 from various organisms. Protein sequence alignment was performed by ClustalW and the result is shown by Colorfy.
(1.77 MB TIF)
