# Supplementary figures and images for: Synthesizing and Salvaging NAD+: Lessons Learned from Chlamydomonas reinhardtii
Source: PLoS Genet. 2010 Sep 9;6(9):e1001105. doi: 10.1371/journal.pgen.1001105 (PMC2936527; doi:10.1371/journal.pgen.1001105)

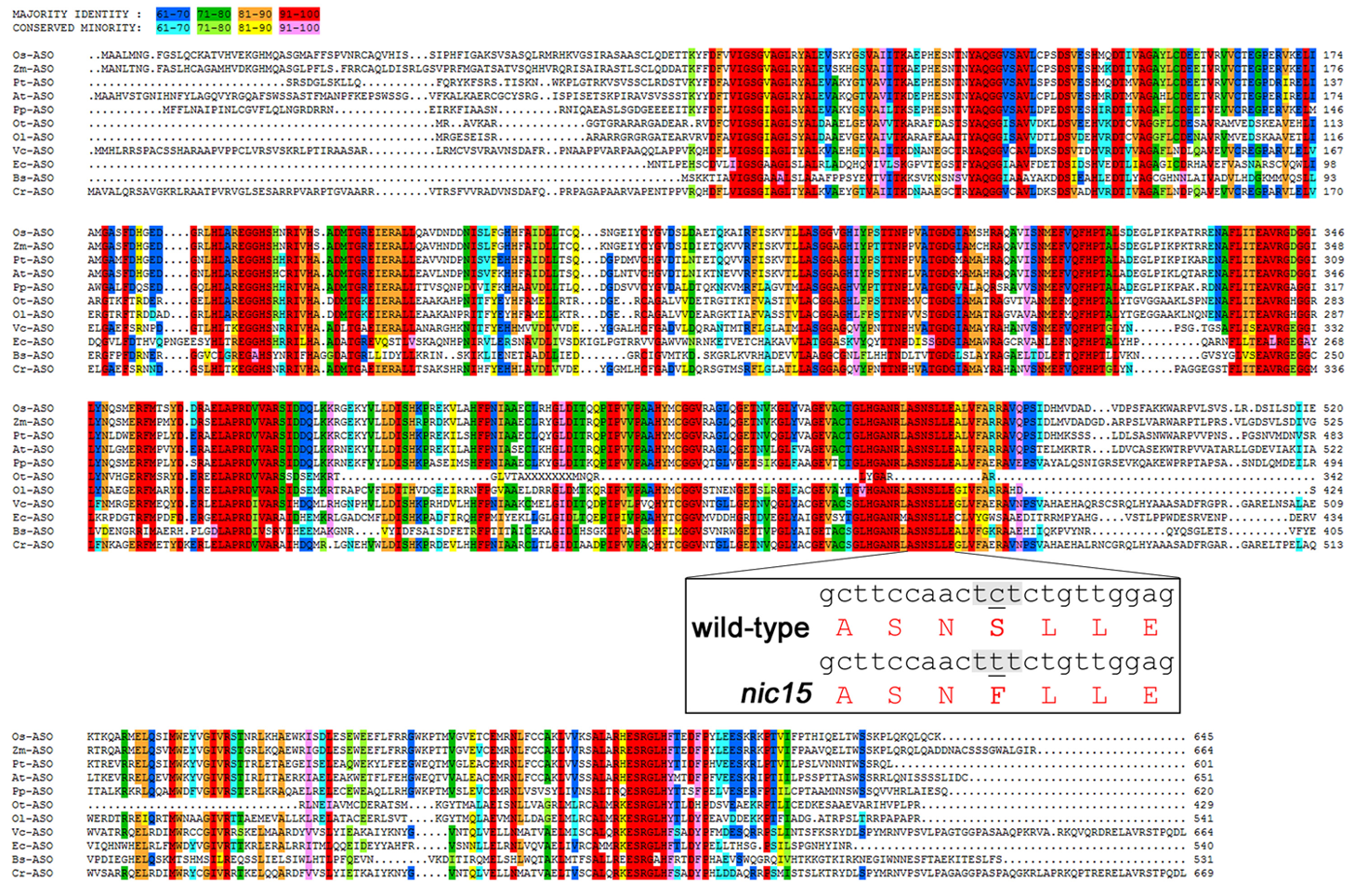

Supplement: Figure S1 — The nic15-1 mutant strain has a missense mutation in aspartate oxidase (ASO). Protein sequence alignment of ASO from various organisms was performed by ClustalW [83] and the result is shown using Colorfy. Colorfy groups the twenty amino acids into eight separate conservation groups ({G, A}, {V, L, I}, {F, Y, W}, {C, M}, {K, R, H}, {D, E, N, Q}, {S, T}, {P}). Percentage composition is defined on a per column basis and categorized as Majority Identity, Conserved Minority or Insufficient Conservation. A column is Majority Identity when at least 61% of the amino acids in that column are identical. A column is Conserved Minority when at least 61% of the amino acids in that column belong to the same conservation group and no amino acid makes up more than 60% of that column. A column is Insufficient Conservation when its composition fails to satisfy any of the prior two conditions. Columns are colored based on percentage composition (Blue: 61 to 70; Green: 71 to 80; Gold: 81–90; Red: 91 to 100). Colors codes are divided into two shades, dark and light. A Majority Identity column can have up to two colors in the column: dark to indicate the positions of the identity amino acid and light to indicate positions of amino acids belonging to the same group as the identity amino acid. A Conserved Minority is colored the light color of the corresponding percentage composed of the majority amino acid group. Columns categorized as Insufficient Conservation are left uncolored. If a column satisfies Majority Identity at a lower percentage and Conserved Minority at a higher percentage, the Majority Identity categorization takes precedence and the column is colored per the Majority Identity percentage. The nucleotide sequences and the corresponding protein sequences around the mutation point for wild-type and nic15-1 are shown in the box. The mutated nucleotide is underlined and the changed amino acid is shown in bold. The color of individual amino acids corresponds to their identity per [file pgen.1001105.s001.tif]

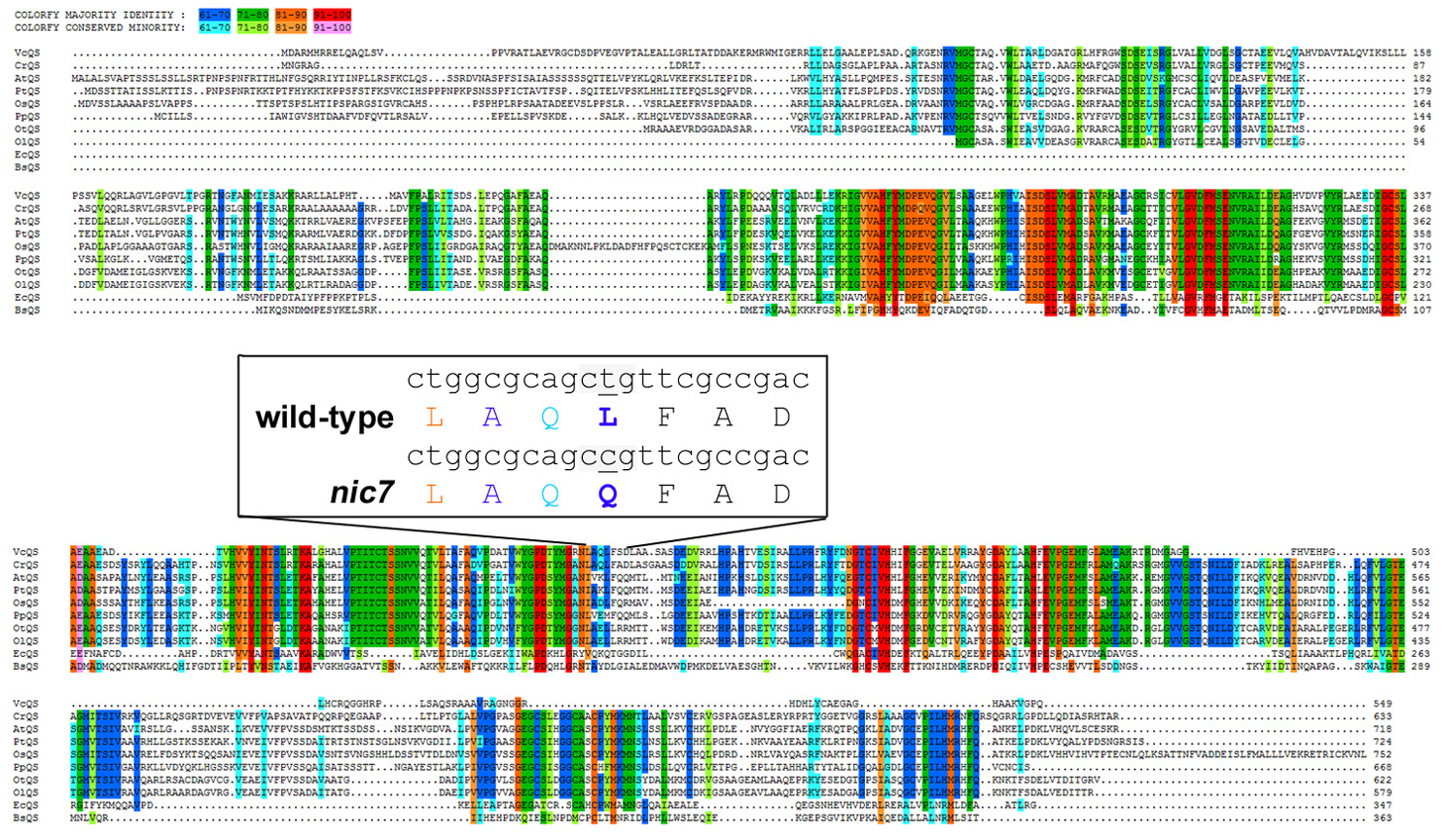

Supplement: Figure S2 — The nic7-1 mutant strain has a missense mutation in quinolinate synthetase (QS). Protein sequence alignment of QS from various organisms was performed by ClustalW and the result is shown by Colorfy. The nucleotide sequences and the corresponding protein sequences around the mutation point for wild-type and nic7-1 are shown in the box. The mutated nucleotide is underlined and the changed amino acid is shown in bold. The color of individual amino acids corresponds to their identity percentages among different organisms. (2.26 MB TIF) [file pgen.1001105.s002.tif]

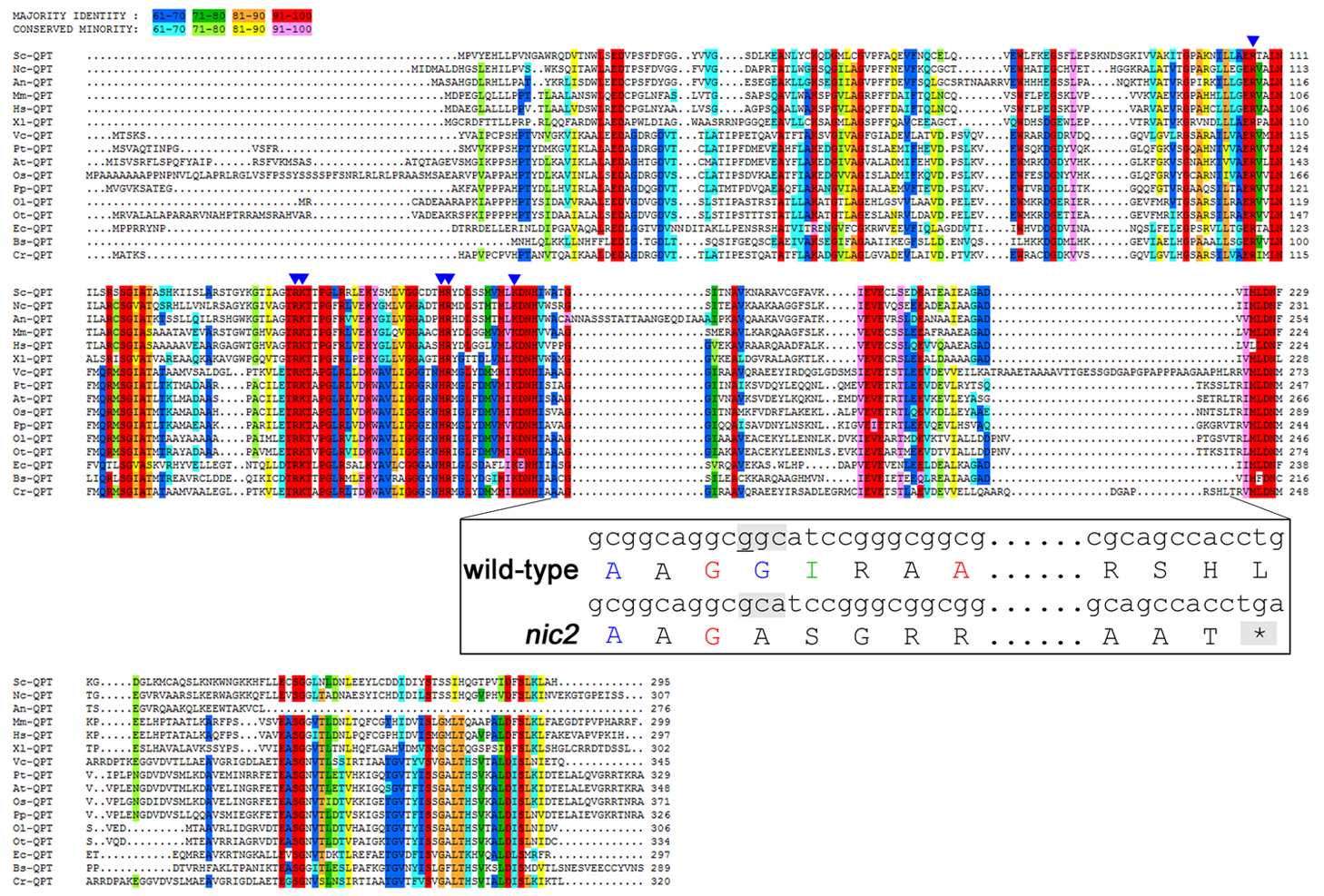

Supplement: Figure S3 — The nic2-1 mutant strain has a deletion of a single nucleotide in quinolinate phosphoribosyltransferase (QPT). Protein sequence alignment of QPT from various organisms was performed by ClustalW and the result is shown by Colorfy. The conserved quinolinate-binding sites are indicated by blue reverse triangles. Partial nucleotide and the corresponding protein sequences for wild-type and nic2-1 are indicated in the box. The deleted nucleotide is underlined in the wild-type. The deletion causes a frame shift that results in a stop codon (*) at amino acid 240. An, Aspergillus nidulans; Hs, Homo sapiens; Mm, Mus musculus; Nc, Neurospora crassa; Sc, Saccharomyces cerevisiae; Xl, Xenopus laevis. (2.13 MB TIF) [file pgen.1001105.s003.tif]

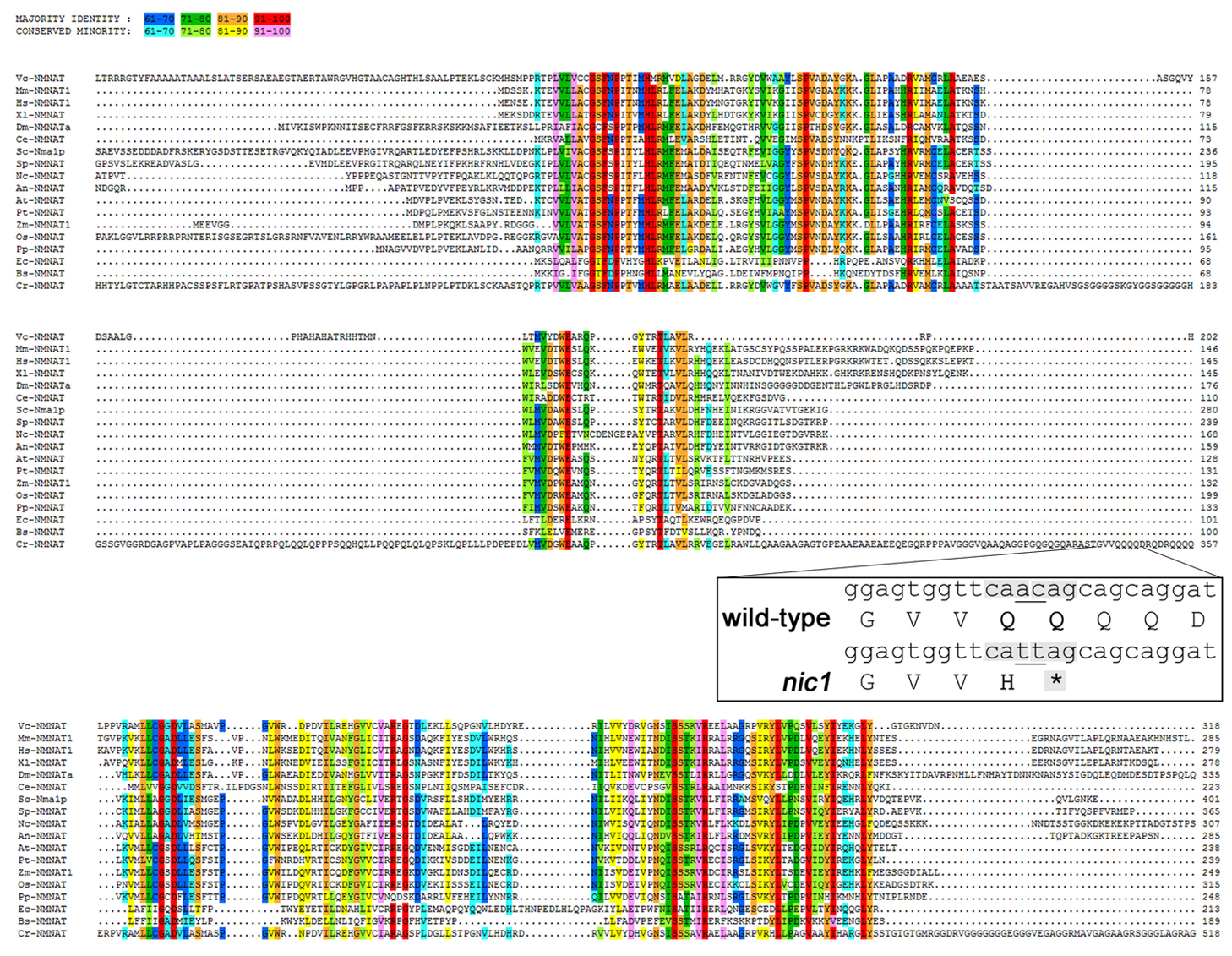

Supplement: Figure S4 — The nic1-1 mutant strain contains a premature stop codon in nicotinamide/nicotinate mononucleotide adenylyltransferase (NMNAT). Protein sequence alignment of NMNAT from various organisms was performed by ClustalW and the result is shown by Colorfy. Partial nucleotide and the corresponding protein sequences for wild-type and nic1-1 are indicated in the box. The mutated nucleotides are underlined, and gray boxes indicate the codons. The amino acid changes are indicated by bold letters. The asterisk indicates a stop codon. Ce, Caenorhabditis elegans; Dm, Drosophila melanogaster; Sp, Schizosaccharomyces pombe. (2.50 MB TIF) [file pgen.1001105.s004.tif]

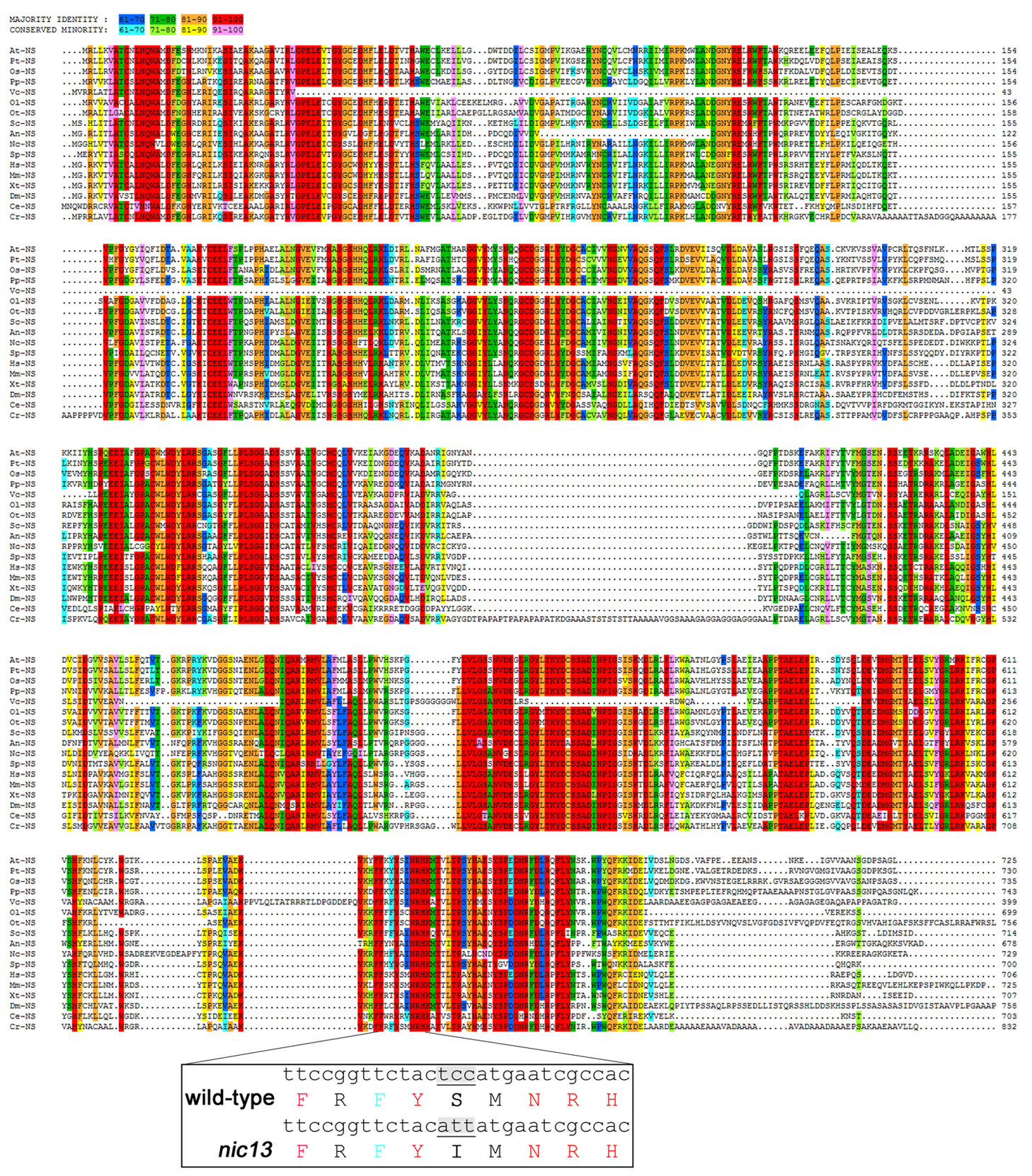

Supplement: Figure S5 — The nic13-1 mutant has a missense mutation in NAD+ synthase (NS). Protein sequence alignment of NS from various organisms was performed by ClustalW and the result is shown by Colorfy. Partial nucleotide and the corresponding protein sequences for wild-type and nic13-1 are indicated in the box. The mutated nucleotides are underlined and the mutated amino acid is indicated by bold letters. (4.63 MB TIF) [file pgen.1001105.s005.tif]

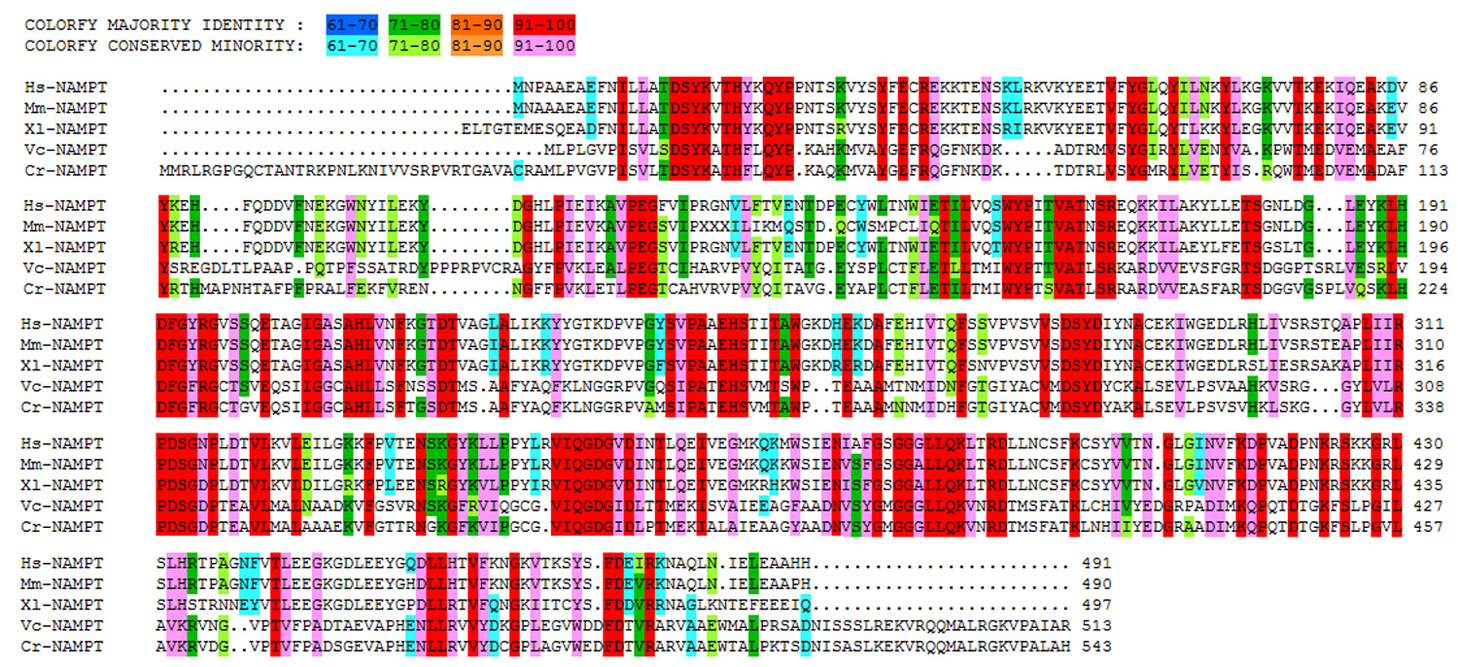

Supplement: Figure S6 — Sequence alignment of nicotinamide phosphoribosyltransferase (NAMPT) from various organisms. Protein sequence alignment of NAMPT was performed by ClustalW and the result is shown by Colorfy. (1.93 MB TIF) [file pgen.1001105.s006.tif]

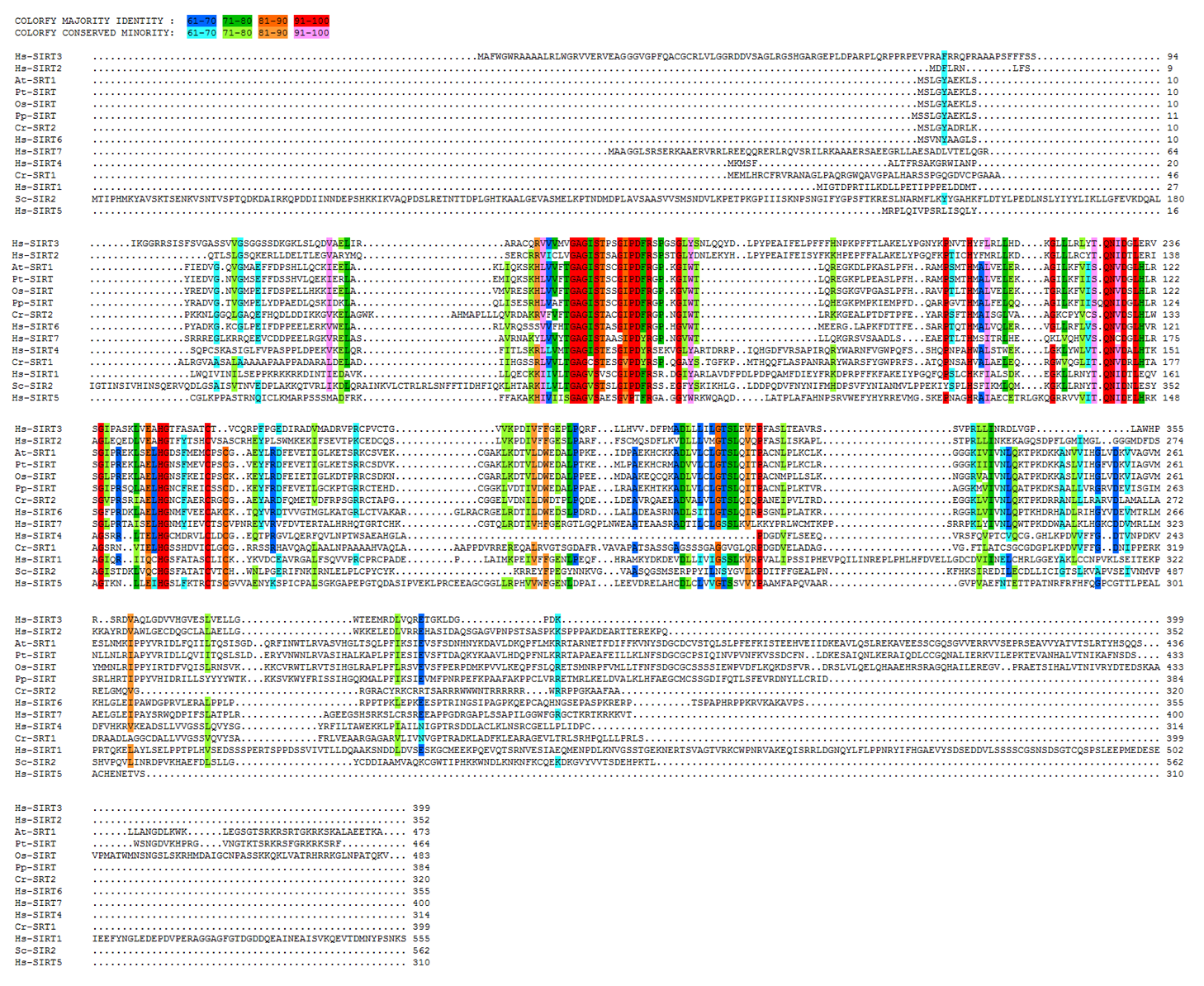

Supplement: Figure S7 — Sequence alignment of SIRT/Sir2 from various organisms. Protein sequence alignment was performed by ClustalW and the result is shown by Colorfy. (1.77 MB TIF) [file pgen.1001105.s007.tif]
